# Electric-Field-Induced Second Harmonic Generation Nonlinear Optic Response of A_4_ β-Pyrrolic-Substituted Zn^II^ Porphyrins: When Cubic Contributions Cannot Be Neglected

**DOI:** 10.1021/acs.inorgchem.0c00451

**Published:** 2020-05-15

**Authors:** Gabriele Di Carlo, Maddalena Pizzotti, Stefania Righetto, Alessandra Forni, Francesca Tessore

**Affiliations:** † Department of Chemistry, University of Milan, INSTM Research Unit, Via C. Golgi 19, 20133 Milano, Italy; ‡ Istituto di Scienze e Tecnologie Chimiche “G. Natta” (SCITEC), Via C. Golgi 19, 20133 Milano, Italy

## Abstract

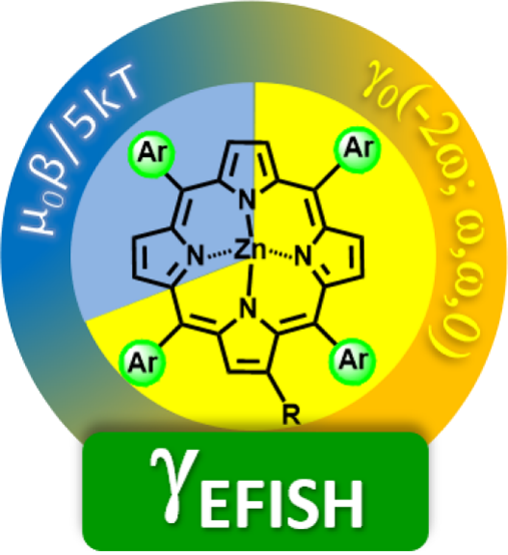

In this work, we have prepared a series of A_4_ Zn^II^ porphyrins, carrying in the β-pyrrolic-position one or two π-delocalized ethynylphenyl moieties with a −NO_2_ acceptor or a −NMe_2_ donor pendant, and measured their second-order NLO response in CHCl_3_ solution at 1907 nm via the electric-field-induced second harmonic generation (EFISH) technique. For some of these compounds, we have recorded an unexpected sign and/or absolute value of μβ_1907_. Since their sterically hindered A_4_ structure should ensure the lack of significant aggregation processes in solution, we explain such anomalous EFISH results by invoking a non-negligible contribution of the electronic cubic term γ(−2ω; ω, ω, 0) to γ_EFISH_, as supported by a qualitative evaluation of the third-order response through the measure of the cubic hyperpolarizability (γ_THG_) and by computational evidence.

## Abstract 2

If the polarity of β-substituted Zn^II^ porphyrins is low, then the contribution of the third-order term γ_0_(−2ω; ω, ω, 0) to γ_EFISH_ cannot be neglected.

## Introduction

Coordination and organometallic compounds, due to the presence of a metal center, offer in comparison to organic chromophores some interesting additional electronic features acting on their second-order nonlinear optical (NLO) response, such as rather strong charge transfer (CT) transitions (ligand to metal and metal to ligand), which can be tuned by working on the oxidation state of the metal and on its coordination sphere. In particular, metal complexes of macrocyclic ligands with a large π electronic system such as porphyrins have been largely investigated, because when their structures are characterized by a specific asymmetric push–pull arrangement a significant directional charge transfer process involving π-polarizable linkers is produced by connecting the donor−π–acceptor system, and significant values of the quadratic hyperpolarizability β are achieved (which is the figure of merit of the second-order NLO response).^1−3^ Moreover, the porphyrin ring is a flexible electronic system, where electron-rich (β-pyrrolic) and electron-poor (*meso*) carbon atoms can be identified.^2^

Interestingly some of us reported for the first time an ambivalent donor/acceptor character of asymmetric monosubstituted porphyrin systems, when connected in the *meso* or β position of the ring to an organic π-delocalized acceptor or donor substituent, respectively.^4,5^

The second-order NLO response of molecular species can be measured in solution by different techniques, such as hyper-Rayleigh scattering (HRS),^6^ Stark effect,^7^ solvatochromism,^3,8^ and electric-field-induced second harmonic generation (EFISH).^9−11^ The EFISH technique is used particularly for the determination of the quadratic hyperpolarizability β of asymmetric dipolar chromophores with an evident push–pull structure, through eq 1:
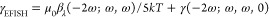
1γ_EFISH_ is the sum of a purely electronic cubic contribution γ(−2ω; ω, ω, 0) (which is a third-order term at the frequency ω of the incident light) and of a quadratic dipolar orientational contribution μ_0_β_λ_(−2ω; ω, ω)/5*kT*, where μ_0_ is the ground-state molecular dipole moment and β_λ_ the projection along the dipole moment direction of the vectorial component β_vec_ of the quadratic hyperpolarizability tensor, when working with the incident wavelength λ.^9^ For the molecules usually investigated by the EFISH technique, the third-order contribution γ(−2ω; ω, ω, 0) is considered to be smaller than the quadratic dipolar orientational term. However, for largely π-delocalized macrocyclic chromophores such as asymmetrically monosubstituted metal porphyrins,^12^ phtalocyanines,^13−15^ or porphyrazines^16^ which show significant third-order NLO properties (whose figure of merit is the cubic hyperpolarizability γ_THG_), such simplification must be carefully and critically applied, because the evaluation of the second-order NLO response by the EFISH technique could be affected by a significant error, with the cubic third-order contribution being comparable, at least on the order of magnitude, to the quadratic orientational one (eq 1).

For example, a γ_EFISH_ value of 1.4 × 10^–33^ esu and a γ_THG_ value of 1.6 × 10^–33^ esu were recorded for an A_3_B-type Pt^II^ porphyrin, with a *para*-OMe substituent on the phenyl groups in 5,10,15-*meso*-positions of the ring and a *para*-NO_2_ group on the phenyl in the 20-position,^12^ and comparable γ_EFISH_ (−1.2 × 10^–33^ esu) and γ_THG_(−1.9 × 10^–33^ esu) responses were also displayed by noncentrosymmetric metal-free phthalocyanines with *tert*-butyl and *para*-tolylsulfonyl substituents.^15^ Moreover, when the second-order NLO response obtained by the EFISH technique is characterized by an unexpected sign and/or absolute value of β_λ_, aggregation or other molecular interactions occurring in solution should be considered.

For instance, we evidenced a significant solvent effect on the second-order NLO response in CHCl_3_ solution for the well-investigated 5,15-push–pull *meso*-diaryl Zn^II^ porphyrin chromophore carrying a -NO_2_ group as acceptor and a −NMe_2_ group as donor, whose nature may control the intermolecular acid–base J aggregation between the basic −NMe_2_ group of one chromophore and the Zn^II^ acid center of another.^17^

Moreover, some of us recently reported that 5,15-push–pull *meso*-diaryl Zn^II^ porphyrins with a π-delocalized substituent carrying an acceptor −COOH group are affected by a complex variety of solvent-dependent aggregation processes in solution,^18^ such as acid–base^17^ or dipolar^19^ interactions between adjacent chromophores or solvolysis induced by the solvent.^20^ The observed aggregation effects are closely related to the *trans-*A_2_BC architecture of the Zn^II^ porphyrin. In fact, they are not observed at all when the push–pull system involves the 2,12-β-pyrrolic-positions of an A_4_ Zn^II^ porphyrin.^1,18^ In this latter architecture, the dihedral angles between the aryl rings and the mean plane of the porphyrin core were indeed reported to lie in the range of 73.5–89.1°, thus lowering the overall flatness of the chromophore and inducing a remarkable steric hindrance to the system.^21^

**Figure 1:**
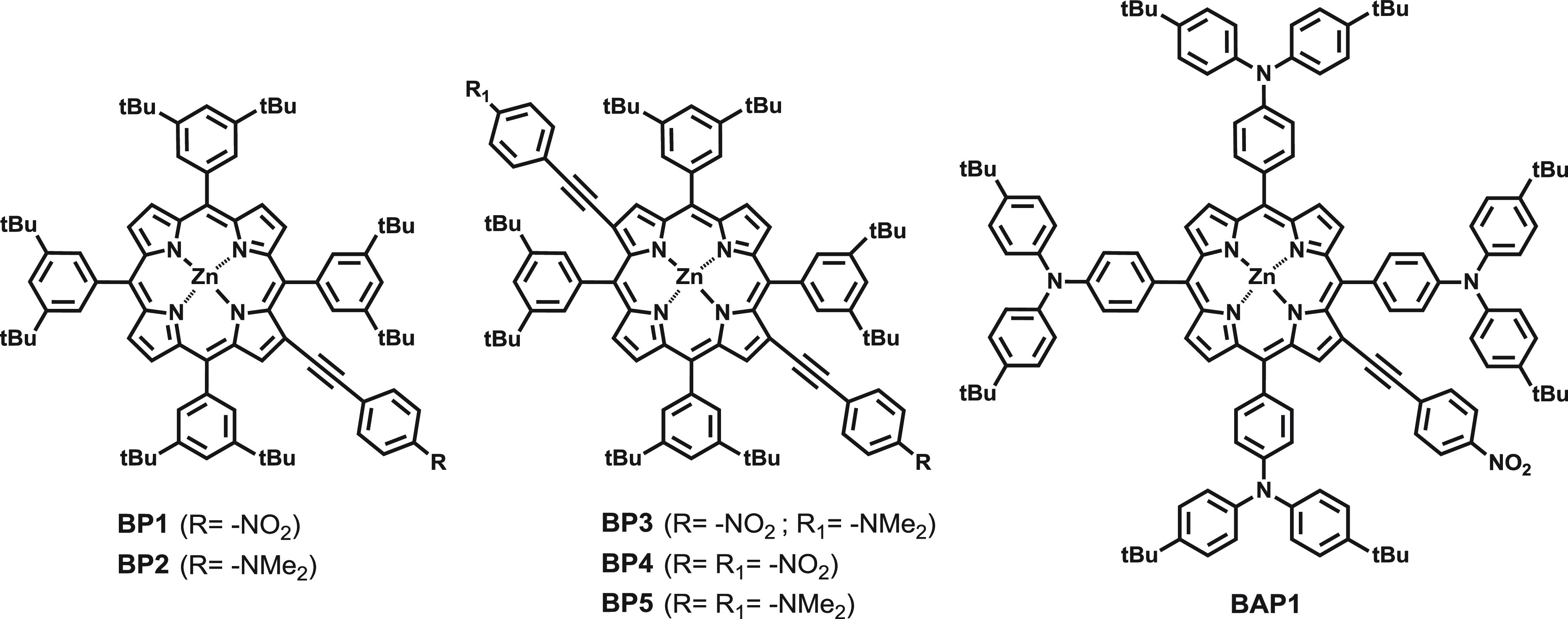
Zn^II^ porphyrins investigated in this work

In this work we add evidence that by safely excluding such secondary effects in solution due to aggregation the third-order contribution to the experimental value of γ_EFISH_ (that is, γ_0_(−2ω; ω,ω,0)) may happen to be not negligible, even for some largely investigated Zn^II^ porphyrinic architectures.^12^ In particular, we focused our attention on some derivatives of 5,10,15,20-tetra(3,5-di-*tert*-butylphenyl)Zn^II^ porphyrin, carrying in the β-pyrrolic-position one or two π-delocalized ethynylphenyl moieties with a −NO_2_ acceptor or a −NMe_2_ donor group (**BP1**–**5**, Figure 1). EFISH measurements in CHCl_3_ solution provided an unexpectedly negative second-order response for some of these compounds. Since their sterically hindered A_4_ structure should ensure the lack of significant aggregation processes in solution,^18,21^ we explain such anomalous EFISH results by the involvement of third-order contributions, also supported by a qualitative evaluation of the third-order response through the measure of the cubic hyperpolarizability γ_THG_, together with computational evidence. In addition, to investigate the effect of an increase of the electron density and of the π-delocalization of the porphyrin core, we studied the NLO properties of the new chromophore ****BAP1**** (Figure 1) by the determination of both γ_EFISH_ and γ_THG_. **BAP1** was first synthesized by some of us^22^ as a dye for dye-synthesized solar cells and has a peculiar and nonclassical 4D−π-1A push–pull electronic structure, due to the four π-delocalized strong donor substituents in the 5,10,15,20-*meso*-positions of the ring.

## Experimental Section

### Materials and Methods

^1^H NMR spectra were recorded on a Bruker Advance DRX-400 in pure CDCl_3_. Due to the higher solubilities of A_4_ β-substituted Zn^II^ porphyrins in comparison to those of the *trans*-A_2_BC analogues, neither the addition of pyridine-*d*_5_ to CDCl_3_ nor the use of expensive THF-*d*_8_ was necessary to acquire well-resolved spectra in the 10^–2^–10^–3^ M concentration range. Mass spectra were obtained with a Bruker-Daltonics ICR-FTMS APEX II with an electrospray ionization source or on a VG Autospec M246 magnetic mass spectrometer with a LSIMS ionic source. Elemental analyses were carried out with a PerkinElmer CHN 2400 instrument in the Analytical Laboratories of the Department of Chemistry at the University of Milan. Electronic absorption spectra were recorded in CH_2_Cl_2_ solution (concentration range of 10^–6^–10^–5^ M) at room temperature on a Shimadzu UV 3600 spectrophotometer. Details on the synthesis of **BP1**–**5**, **BAP1**, and their precursors, including mass spectrometry data, elemental analyses, and ^1^H NMR data and spectra, are reported in Figures S1–S6.

### EFISH and THG Measurements

The second-order NLO responses of chromophores **BP1**–**5** and **BAP1** were measured by the EFISH technique,^9−11^ using a prototype apparatus made by SOPRA (France) and working with a 1907 nm incident wavelength. For each chromophore, measurements were performed on freshly prepared solutions in CHCl_3_ at a concentration of 10^–3^ M. For **BAP1**, a measurement was also made at a concentration of 5 × 10^–4^ M.

The 1907 nm laser incident wavelength was chosen because its second harmonic (at 953 nm) is far enough from the absorption bands of the chromophores in CHCl_3_ (λ_max_ of the B band in the range 420–460 nm and of the Q bands in the range of 560–615 nm; see Table 1) to avoid possible enhancement of the second-order NLO response due to resonance effects. The incident beam was obtained by Raman shifting of the 1064 nm emission of a Q-switched Nd:YAG laser in a high-pressure hydrogen cell (60 bar). A liquid cell with thick windows in the wedge configuration was used to obtain the Maker fringe pattern originated by the harmonic intensity variation as a function of the liquid cell translation. In the EFISH experiments, this incident beam was synchronized with a direct current field applied to the solution, with 60 and 20 ns pulse duration, respectively, in order to break its centrosymmetry. The comparison of the harmonic signal of the chromophore solution with that of the pure solvent allowed the determination of its second-order NLO response (assumed to be real because the imaginary part was neglected).

**Table 1:** Electronic Absorption Data of **BP1**–**5** and **BAP1** in CH_2_Cl_2_ Solution

compound	B bands λ_max_, nm (log ε)	Q_α_ and Q_β_ bands λ_max_, nm (log ε)
**BP1**	435 (5.44)	561 (4.45)
	458 (sh)	599 (4.30)
**BP2**	432 (5.25)	559 (4.26)
		595 (4.04)
**BP3**	443 (5.05)	571 (4.23)
		612 (4.35)
**BP4**	450 (5.02)	572 (4.11)
	464 (5.09)	614 (4.28)
**BP5**	426 (5.27)	572 (4.52)
	485 (sh)	611 (4.63)
**BAP1**	423 (5.02)	567 (4.33)
	461 (5.13)	615 (4.26)

The γ_EFISH_ values reported in Table 3 are the mean values of 12 successive measurements performed on the same sample. All experimental EFISH μ_0_β_1907_ values are defined according to the “phenomenological” convention.^23^

THG experiments were carried out in 10^–3^ M CHCl_3_ solution on the same apparatus used for EFISH experiments but without applying an electric field,^24^ providing the cubic hyperpolarizability γ_THG_(−3ω; ω; ω, ω). Experimental γ_THG_ values could be affected by resonant enhancement because the Q band absorptions are close to the third harmonic 3ω (635 nm); therefore, these values should be taken in consideration mainly as an order of magnitude.^12^ EFISH and THG experiments were carried out in the Department of Chemistry of the University of Milano (Italy).

### Computational Calculations

Density functional theory (DFT) calculations were performed on all compounds using the Gaussian16 suite of programs.^25^ Geometry optimizations were performed with the 6-311G(d) basis set using the M06 functional,^26^ due to its specific parametrization on organometallic complexes. Excitation energies were computed at TD-B3LYP/6-311g(d) level in dichloromethane, on the basis of previously reported theoretical investigations of analogue porphyrin systems.^18^ Using the same basis set, SHG first hyperpolarizabilities, i.e., the β(−2ω; ω, ω) tensors, were computed within the Coupled Perturbed Kohn–Sham (CPKS) approach at the same frequency (1907 nm) used in the EFISH experiments. The SHG second hyperpolarizabilities, i.e., the γ(−2ω; ω, ω, 0) tensors, were evaluated by finite field technique. The M06-2X functional,^26^ which has been recently recommended for hyperpolarizability calculations of midsize chromophores,^27^ was adopted for both β and γ calculation. The same functional was used for determining the dipole moments μ_0_. A pruned (99,590) grid was selected for computation and use of two-electron integrals and their derivatives. To get a meaningful comparison with the experimental data, the scalar quantities β_||_ and γ_||_ were derived from the full tensors β and γ, respectively; β_||_ corresponds to 3/5 times β_λ_, the projection along the dipole moment direction of the vectorial component of the β tensor, that is, β_||_ = (3/5) Σ_*i*_(μ_*i*_β_*i*_)/μ, where β_*i*_ = (1/5)Σ_*j*_(β_*ijj*_ + β_*jij*_ + β_*jji*_).^28,29^ γ_||_ is related to the tensor components according to the following: γ_||_ = (1/15) [3(γ_*xxxx*_ + γ_*yyyy*_ + γ_*zzzz*_) + 2(γ_*xxyy*_ + γ_*xxzz*_ + γ_*yyzz*_ + γ_*yyxx*_ + γ_*zzxx*_ + γ_*zzyy*_) + (γ_*xyyx*_ + γ_*xzzx*_ + γ_*yzzy*_ + γ_*yxxy*_ + γ_*zxxz*_ + γ_*zyyz*_)].^28^

## Results and Discussion

### Synthesis of A_4_ β-Substituted Zn^II^ Porphyrins

**Scheme 1:**
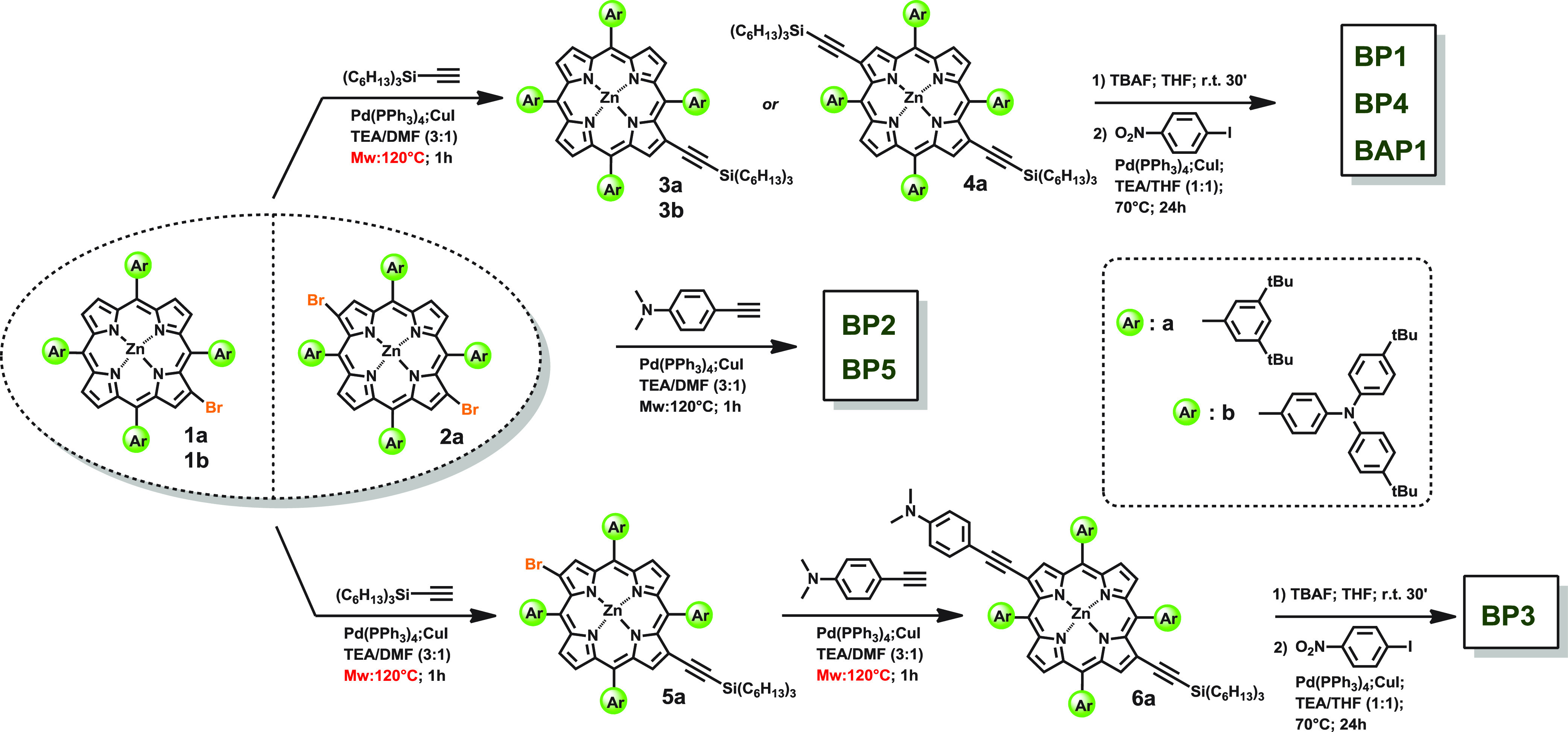
Schematic Synthetic Procedure for **BP1**–**5** and **BAP1**

Porphyrins with one ethynyl substituent on β-pirrolic position are well-studied, and several synthetic procedures are reported in literature. The examples of porphyrins disubstituted at the pyrrolic sites to produce a push–pull systems are very rare,^30^ by antipodal insertion of an electron-donor at one end and electron-acceptor at the other end. To get porphyrins with such geometries, the crucial strategy lies in the regioselective bromination of the porphyrin core at the β-position as previously reported by some of us.^21^ Thus, the synthetic strategy (Scheme 1) to obtain the chromophores investigated in this contribution required to adopt building-blocks as 2-bromo (**1a** and **1b**) and 2,12-dibromo (**2a**) functionalized Zn^II^ porphyrins,^21,22,31^ for the preparation of the monosubstituted porphyrins (**BP1**, **BP2**, and ****BAP1****) and of the disubstituted ones (**BP3**, **BP4**, and ****BP5****), respectively. To further introduce the selected nitro- and amino-based pendants at the periphery of the porphyrin core, the Pd/catalyzed microwave-assisted Sonogashira coupling reaction^31^ between proper ethynyl substituents and the brominated intermidiates, **1a** and **1b**, was explored. While an effective insertion at the β-positions of 4-ethynyl-*N*,*N*-dimethylaniline smoothly provided desired **BP2** and **BP5** (Scheme 1), in contrast, 1-ethynyl-4-nitrobenzene barely reacted in those conditions to get desired chromophores **BP1**, **BP3**, **BP4**, and **BAP1**. As a result, a multistep procedure was designed to push up the functionalization yield of pyrrolic carbons with the nitro terminal pendant. In order to overcome the reactivity barrier related to the direct functionalization of bromo porphyrins with nitro based acetylenic substituents, silyl-protected acetylenic terminal linkers were first introduced into 2 and 12 positions (**3a**, **3b**, **4a**, **5a**, and **6a** in Scheme 1) by microwave-enhanced Sonogashira coupling reaction. The subsequent tetrabutylammonium fluoride (TBAF) treatment almost quantitatively yielded the unprotected acetylenic terminal intermediates which easily reacted, by a classic thermal Sonogashira coupling reaction, with 1-iodo-4-nitrobenzene to successfully give desired products **BP1**, **BP3**, **BP4**, and **BAP1**. Thus, as depicted in Scheme 1 and detailed in the Supporting Information, the designed multistep approach allowed us to efficiently synthesize monosubstituted **BP1**, **BP2** and **BAP1**, symmetric 2,12-disubstituted **BP4** and **BP5**, and 2,12 asymmetric disubstituted **BP3** porphyrinic chromophores.

### UV–Vis Absorption Spectroscopy

**Figure 2:**
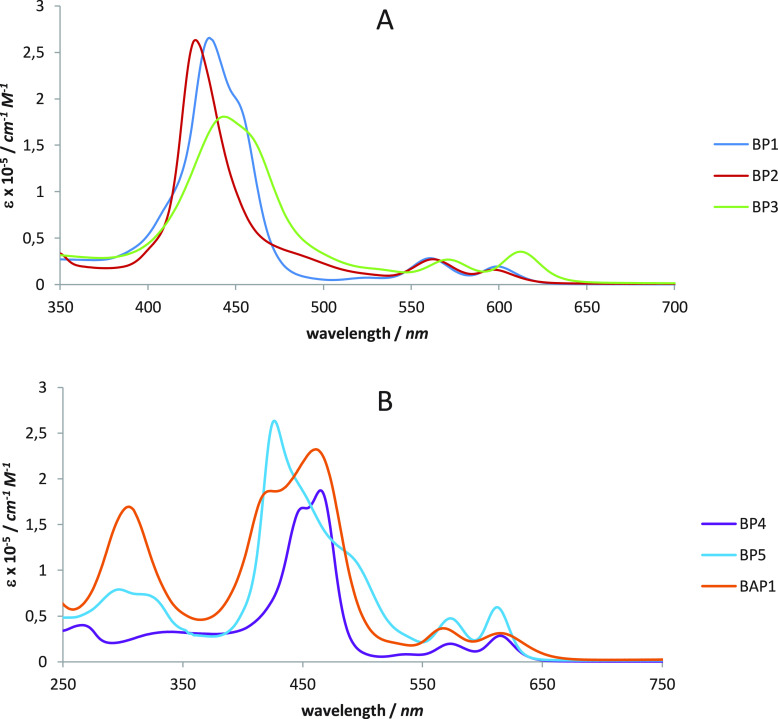
(a) Electronic absorption spectra of BP1, **BP2**, and **BP3** in CH_2_Cl_2_. (b) Electronic absorption spectra of **BP4**, **BP5**, and **BAP1** in CH_2_Cl_2_.

The UV–vis absorption spectra of **BP1**–**5** and **BAP1** in CH_2_Cl_2_ solution at 1.0 × 10^–5^ M concentration are reported in Figure 2a,b, while the corresponding experimental data are given in Table 1.

The UV–vis spectra of **BP1** and **BP2** (Figure 2a) show the typical pattern expected for A_4_ β-pyrrolic-monosubstituted Zn^II^ porphyrins on the basis of the “four-orbital model” proposed by Gouterman,^32^ with a very strong (ε ∼ 10^5^ M^–1^ cm^–1^) absorption B band at about 430 nm, due to the S_0_ → S_2_ transition (from the ground to the second excited state) and two weaker (ε ∼ 10^4^ M^–1^ cm^–1^) Q bands in the range of 500–600 nm, due to the S_0_ → S_1_ transition (from the ground to the first excited state). Q_α_ is the band at higher energy, and Q_β_ is that at lower energy.^33^

In the case of **BP1**, a weaker band appears as a shoulder at lower energy of the B band, as a result of the electron-withdrawing properties of the −NO_*2*_ group. As reported for the corresponding Zn^II^ porphyrin with a cyanoacrylic moiety, strong electron acceptors induce a remarkable perturbation to the “four-orbital model”, breaking the degeneracy of the LUMO and LUMO+1 orbitals and stabilizing the LUMO energy level. Moreover, the LUMO+2 and LUMO+1 orbitals become nearly degenerate, with a decrease of the HOMO–LUMO energy gap and the formation in the electronic absorption spectrum of a red-shifted shoulder of the B band.^31,34,35^ However, when the ethynylphenyl moiety in β-position carries a −NMe_2_ donor group as in **BP2**, the B band is symmetrical, as reported to occur when the aryls in the 5,10,15,20-*meso*-positions are simple phenyl groups.^4^ In addition, both the B and the Q bands show a slight hypsochromic shift in comparison to BP1.

The asymmetric disubstitution in 2,12-β-pyrrolic-positions with ethynyphenyl fragments carrying a donor and an acceptor group (as in **BP3**) leads to a slight lowering of the B band intensity, in addition to an increase of its bandwidth and a sizable bathochromic shift, particularly for the Q bands (Figure 2a). These spectroscopic features are in agreement with an increased π-conjugation and the push–pull character of the molecule, resulting in a lower HOMO–LUMO gap (Table 2).

**Table 2:** Correlation between Experimental λ_max_ of the Q_β_ Bands and the HOMO–LUMO Gap and Contribution of the HOMO–LUMO Transition to the Q_β_ Bands of **BP1**–**5** and **BAP1** as Computed at the M06-2*X*/6-311G(d) Level in Dichloromethane

compound	experimental Q_β_ band λ_max_, nm	HOMO–LUMO gap, eV	contribution of the HOMO–LUMO transition to the Q_β_ band
**BP1**	605	2.40	89%
**BP2**	573	2.60	78%
**BP3**	643	2.23	89%
**BP4**	624	2.34	90%
**BP5**	605	2.46	89%
**BAP1**	694	2.19	34%^a^
	639^b^		56%^b^,^c^

a Contribution of 61% HOMO–1–LUMO.

b Referred to as the Q_α_ band.

c Contribution of 31% HOMO–1–LUMO.

The electronic spectra of symmetric disubstituted Zn^II^ porphyrins **BP4** and **BP5** show an interesting doubling of the B band, which is more pronounced in the presence of electron-acceptor −NO_2_ groups (Figure 2b). Furthermore, we observed a significant redshift of the Q bands with respect to those of corresponding asymmetric monosubstituted Zn^II^ porphyrins **BP1** and **BP2**. Finally, in the spectra of disubstituted complexes the intensity of the Q_β_ band is higher than that of the Q_α_ band, while the opposite behavior is noticed for monosubstituted complexes (Q_α_ > Q_β_). All these features are in support of the perturbation of the electronic properties of the porphyrin core induced by π-delocalized ethynylphenyl moieties.^4^

The replacement in the 5,10,15,20-*meso*-positions of the core of 3,5-di-*tert*-butylphenyl groups with bulky and strongly donor bis(4-*tert*-butylphenyl)anilines leads to dramatic changes of the UV–vis spectrum. If we compare the spectra of **BP1** (Figure 2a) and **BAP1** (Figure 2b), then a doubling of the B band appears for the latter, with a remarkable increase of bandwidth and a sizable red-shift of the Q bands. Interestingly, when the −NO_2_ acceptor substituent connected by an ethynylphenyl linker to the β-pyrrolic-position was swapped for a cyanoacylic group, the spectrum showed only one single, although broad, B band.^22^ This suggests that the peculiar features of the B band of **BAP1** are induced by the −NO_2_ group.

The differences between **BP1** and **BAP1** cannot be ascribed to aggregation phenomena, since aggregation in solution is not relevant for sterically hindered A_4_ β-pyrrolic-substituted Zn^II^ porphyrins.^18,21^ Rather, they can be due to the peculiar electronic structure of the 4D-π-1A architecture of **BAP1**.

However, to further exclude the presence of any aggregation phenomenon, UV–vis spectra in CH_2_Cl_2_ at different concentrations in the range of 10^–6^–10^–5^ M were recorded (Figures S7–S12). As expected, neither deviation from the Lambert–Beer law, nor shift of the wavelength maximum or of the B or Q bands was detected by increasing concentration, different from that reported for *trans*-A_2_BC type Zn^II^ porphyrins.^17^ The UV–vis evidence is supported also by the well-resolved signals in the ^1^H NMR spectra, acquired at a concentration 3 orders of magnitude higher (10^–2^–10^–3^ M) (see the “Materials and Methods” section and Figures S1–S6).

### DFT Calculations

**Figure 3:**
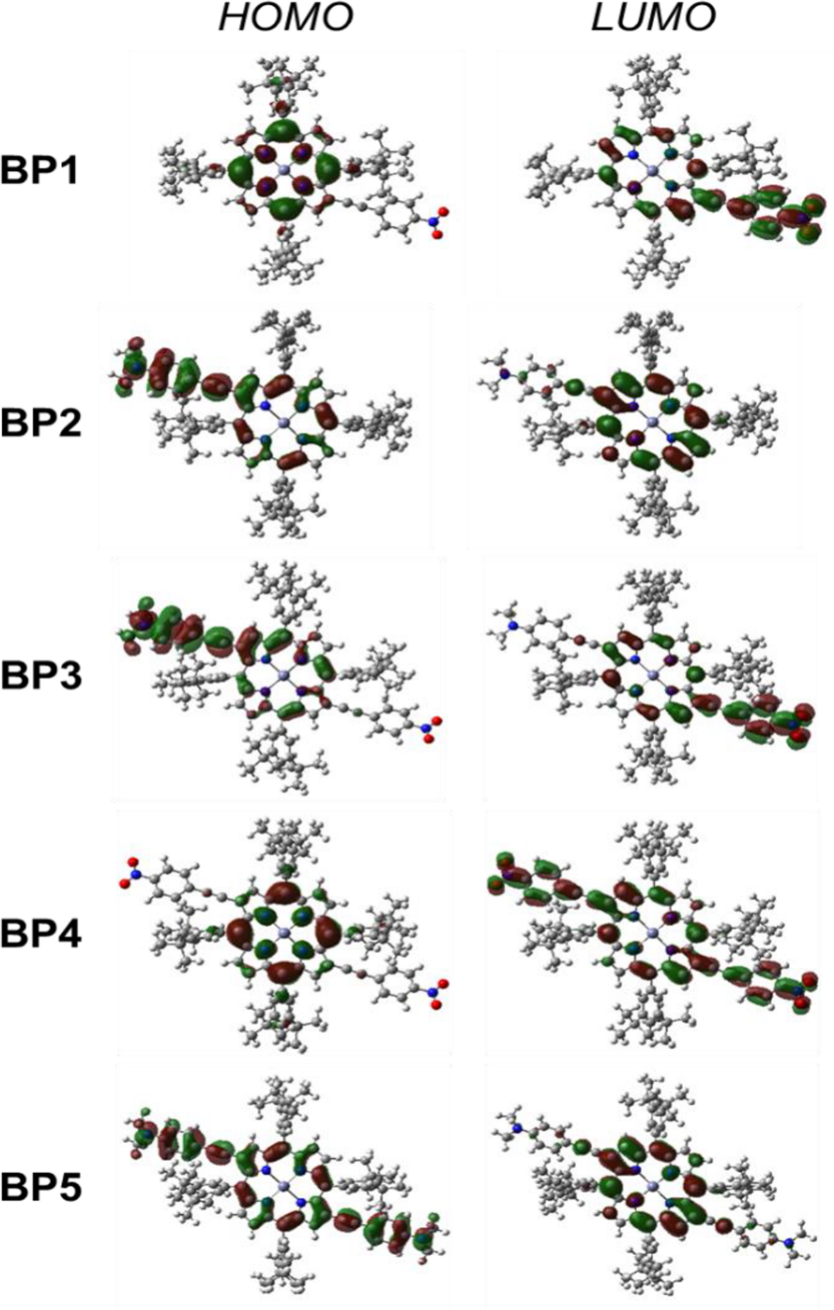
Isodensity plots of HOMO and LUMO of **BP1**–**5** (isosurface values: 0.02).

In our previous investigation^4^ on A_4_ β-pyrrolic-monosubstituted Zn^II^ porphyrins, with a π substituent structurally similar to that of **BP1** and **BP2** but carrying a phenyl group in the 5,10,15,20-*meso*-positions of the ring, we evidenced for the first time (by UV–vis electronic absorption spectroscopy, solvatochromism and voltammetry) a charge transfer process from the porphyrin core to the π-substituent in the β-position when this latter carries a strong −NO_2_ acceptor group or from the π-substituent in the β-position to the porphyrin core, if the former carries a strong −NBu_2_ donor group. The ambivalent donor–acceptor character of this kind of β-substituted 5,10,15,20*-*tetraphenyl Zn^II^ porphyrin was also supported by second-order NLO measurements based on the EFISH technique. Later, Anderson, Clays, and co-workers^36^ confirmed that the porphyrin core can behave as an acceptor by hyper-Rayleigh scattering measurements on a system carrying an electron donor attached to one *meso*-position of the ring. However, neither investigation took into consideration the possible effects of aggregation in solution on the second-order NLO responses. Moreover, no theoretical evidence to support the proposed charge transfer process from or to the porphyrin core was given. Therefore, in the present work, we have carried out a DFT investigation on **BP1**–**5** and **BAP1**. The DFT HOMO–LUMO energy gaps of **BP1**–**5** and **BAP1** are reported in Table 2, and the HOMO and LUMO isodensity plots are given in Figure 3.

The plots clearly confirm our previous qualitative suggestions. For **BP1**, the HOMO → LUMO transition is characterized by a significant charge transfer from the porphyrin core to the – C≡C–C_6_H_4_–NO_2_ acceptor substituent linked to the β-pyrrolic-position. For **BP2**, an opposite electron transfer occurs from the -C≡C–C_6_H_4_–NMe_2_ donor substituent in the β-pyrrolic-position to the porphyrin core. Interestingly, DFT calculations suggest for **BP1** a more significant electron transfer process than that for **BP2** (Table 2), suggesting that the porphyrin core behaves better as a donor than as an acceptor.

The new push–pull Zn^II^ porphyrin, **BP3**, involving the 2,12-β-pyrrolic-positions of the porphyrinic ring,^21^ is characterized by a significant electron transfer from the donor acetylenic substituent to the acceptor substituent, as confirmed by its isodensity plot (Figure 3), with a limited involvement of the electron density of the porphyrin core, which seems to play the role of a simple bridge of the push–pull electronic system.

For symmetric chromophores **BP4** and ****BP5****, the HOMO → LUMO transition is characterized by an electron transfer which involves the porphyrin core. When the ethynylphenyl moieties in 2,12 carry −NMe_2_ groups (**BP5**), a substituent to the porphyrin core electronic transfer occurs, while in the presence of two −NO_2_ substituents (**BP4**), the electron transfer is from the porphyrin core to the ethynylphenyl fragment. Such DFT data are in agreement with the proposed “electronic softness” of the Zn^II^ porphyrin core with respect to the perturbation introduced by β-pyrrolic substitution with a push or a pull acetylenic system.^4^

The introduction of a significant electronic asymmetry in the porphyrin architecture was confirmed by the DFT-computed ground-state dipole moments (μ, Table 4). The value of 10.6 D for push–pull chromophore **BP3** is quite close to that reported for the well-investigated push–pull Zn^II^ porphyrin carrying in the 5,15-*meso*-positions the same acetylenic substituents (13.9 D).^37^ Dipole moments also confirm a higher charge asymmetry for **BP1** (μ = 7.8 D) than for **BP2** (μ = 2.7 D), in agreement with the proposed lower perturbation of the ground state of the porphyrin core induced by an ethynylphenyl moiety with a −NMe_2_ donor group.^4^ Such lower perturbation is also supported by the HOMO–LUMO energy gap (Table 2). Indeed, it is higher for **BP2** (2.60 eV) than it is for **BP1** (2.40 eV), as expected for a less easy electron transfer when the porphyrin core is substituted in the β-position by a donor acetylenic system.

As far as disubstituted Zn^II^ porphyrin **BP3** is concerned, its HOMO–LUMO energy gap is lower than those of monosubstituted **BP1** and **BP2**, suggesting an improved charge-transfer. However, if it is compared to the HOMO–LUMO gap of the corresponding 5,15-*meso*-disubstituted Zn^II^ porphyrin, then it is higher (2.23 eV vs 2.03 eV), revealing a less efficient conjugation when the push–pull system involves 2,12-β-pyrrolic-positions.

Finally, the contribution of the HOMO–LUMO transition to the Q_β_ band is about 90% for both **BP1** and **BP2**, while for **BP2** it is only 78%, in agreement with the limited acceptor properties of the porphyrin core (Table 2).

To sum up, our DFT investigation evidenced that in A_4_ B-pyrrolic Zn^II^ porphyrins with 3,5-di-*tert*-butylphenyl groups in the 5,10,15,20-*meso*-positions the porphyrin core behaves as a good donor but as a weaker and less efficient acceptor, as expected from the electron-rich nature of the pyrrolic position, first highlighted by Marks and Ratner.^2^

**Figure 4:**
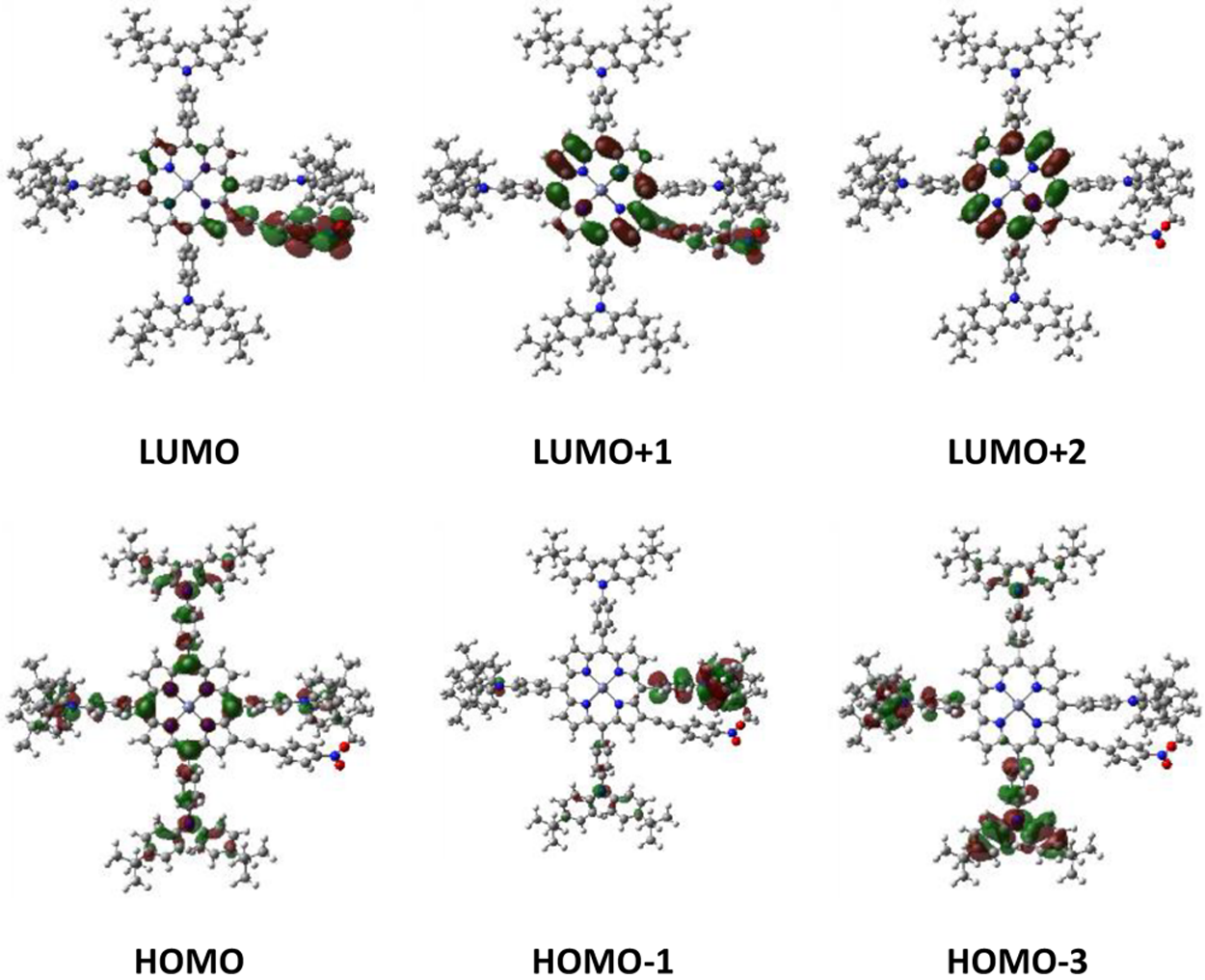
Isodensity plots of frontier orbitals of **BAP1** (isosurface values: 0.02).

In contrast, **BAP1** shows quite different and interesting electronic properties. Indeed, the Q_β_ band of this compound is due for only 34% to the HOMO–LUMO transition, while the (HOMO–1)–LUMO transition becomes relevant with a 61% contribution (Table 2). Looking at the isodensity plots (Figure 4), this transition is mainly associated, different from **BP1**, with an electron transfer from one of the bulky and strongly donor bis(4-*tert*-butylphenyl)anilines in the *meso*-position to the adjacent π-conjugated ethynylphenyl moiety carrying the −NO_2_ acceptor group in the β-pyrrolic-position. The electron density of the porphyrin core is involved only in the less important HOMO–LUMO transition, which has a minor role in the Q_β_ band composition (Figure 4).

Moreover, it is also interesting that in **BAP1** the HOMO energy level is not delocalized on the porphyrin ring, as occurs for **BP1** and **BP4**, but rather is mainly distributed on the four bis(4-*tert*-butylphenyl)anilines substituents in the *meso*-position (Figure 4).

Therefore, in **BAP1** the porphyrin core cannot be considered a classical donor system in the electron transfer process to the −NO_2_-substituted acetylenic moiety in the β-pyrrolic-position, as occurs for **BP1** (Figure 3).

### Experimental and Theoretical Investigation of the Second-Order NLO Properties

EFISH measurements of ****BP1****–**5** and **BAP1** were carried out in CHCl_3_ solution at a concentration of 10^–3^ M with an incident wavelength of 1907 nm and are reported in Table 3. **BAP1** was also tested at 5 × 10^–4^ M, to exclude the presence of aggregation phenomena in solution.

**Table 3:** Experimental μ_0_β_1907_ and γ_EFISH_ Values of **BP1**–**5** and **BAP1** in CHCl_3_ at 1907 nm

compound	μ_0_β_1907_ (× 10^–48^), esu	γ_EFISH_ (× 10^–33^), esu
**BP1**^a^	730	3.5
**BP2**	–320	–2.23
**BP3**	690	3.1
**BP4**	–157	–0.87
**BP5**	–230	–1.15
**BAP1**^b^	–685^c^	–3.6

a γ_THG_ = 1.5 × 10^–33^ esu.

b γ_THG_ = −5.1 × 10^–33^ esu.

c −740 × 10^–48^ esu at 5 × 10^–4^ M.

The μ_0_β_1907_ values were obtained by eq 1, assuming a negligible contribution to γ_EFISH_ of the term γ(−3ω; ω, ω, 0), and for this reason could be overestimated. However, γ(−2ω; ω, ω, 0), cannot be always overlooked, as it was reported for some asymmetrically substituted metal phtalocyanines^15,38,39^ or metal porphyrins with A_4_ or A_3_B architecture.^12^ In particular, this occurs when the order of magnitude of the cubic hyperpolarizability (γ_THG_), which expresses the third-order NLO properties of the molecule, is comparable to that of γ_EFISH_. For this reason, for largely π-delocalized molecules it is sometimes useful to combine EFISH and THG measurements.^40^

Indeed, γ_THG_ values of largely π-delocalized molecular architectures such as metal phtalocyanines^39^ and metal porphyrins^12^ have been often reported to be a fair way to assess the relevance of the third-order term contribution to γ_EFISH_. In particular, γ(−2ω; ω, ω, 0) was considered not negligible when γ_EFISH_ and γ_THG_ differ by less than 5–20%.^40^ Therefore, significant values of γ_THG_ compared with γ_EFISH_ may support a not-trivial contribution of the electronic cubic term in eq 1 to γ_EFISH_.

The quite high DFT values of the ground-state dipole moments (μ_0_) of **BP1** and particularly of **BP3** (Table 4) would suggest for these chromophores a predominant contribution of the dipolar orientational term μ_0_β/5*kT* to γ_EFISH_. In agreement, the μ_0_β_1907_ values for **BP1** and **BP3** (Table 3) are positive and quite high, as for the Zn^II^ porphyrin structurally analogous to **BP1**, but carrying in the 5,10,15,20-*meso*-positions simple phenyl groups instead of the bulkier and slightly donor 3,5-di-*tert*-butylphenyl groups.^4^

**Table 4:** Theoretical μ_0_, β_∥_, μβ_∥_/5*kT*, and γ_∥_ Values of ****BP1****–**5** and **BAP1**

compound	μ_0_, D	β_∥_ (× 10^–30^), esu	μβ_∥_/5*kT* (× 10^–36^), esu	γ_∥_ (× 10^–36^), esu
**BP1**	7.8	72	2720	–773
**BP2**	2.7	60	790	–888
**BP3**	10.6	189	9800	–2139
**BP4**	0.6	3	8	–1514
**BP5**	0.6	4	12	–1971
**BAP1**	6.2	64	1950	–2042

Moreover, CP-DFT calculations *in vacuo* (Table 4) provided, particularly for 2,12-β-disubstituted push–pull chromophore **BP3**, a high and positive value of β_||_, 2.5–3 times higher than those of monosubstituted **BP1**, **BP2**, and **BAP1**, characterized by a lower polarity.

However, the negative μ_0_β_1907_ and γ_EFISH_ values recorded for **BP2** and **BAP1** (Table 3) are totally unexpected. Since they cannot be ascribed to molecular aggregations, due to the significant steric hindrance of the molecular architectures,^18,21^ we tentatively attribute them to a negative contribution of the electronic cubic term γ(−2ω; ω, ω, 0) to γ_EFISH_. In order to support this hypothesis, we have experimentally measured the γ_THG_ values of **BP1** and **BAP1**, as the more emblematic cases in our series of compounds, since they differ only for the aryl groups in the 5,10,15,20-*meso*-positions, but nevertheless display positive (+1.5 × 10^–33^ esu) and negative (−5.1 × 10^–33^ esu) γ_THG_ values, respectively.

According to DFT calculations, providing negative γ_||_ = γ(−2ω; ω, ω, 0) values^28^ for all the porphyrin chromophores here investigated (Table 4), we suggest that the electronic third-order term may overwhelm the positive value of the dipolar orientational term μβ_||_ for symmetric **BP4** and **BP5** and particularly for slightly asymmetric **BP2** chromophores, thus providing a negative γ_EFISH_ value (Table 3).

Such a suggestion is further supported by comparing the negative value of γ_||_ calculated for **BAP1** (Table 4) with the relevant negative value of its measured γ_THG_. The increased π-delocalization of the molecular structure of **BAP1** due to the presence of the four bis(4-*tert*-butylphenyl)anilines as expected enhances the third-order NLO properties,^14,41^ leading to a 2-fold γ_THG_ with respect to that reported for an A_3_B-type Zn^II^ porphyrin (2.2 × 10^–33^ esu).^12^ Furthermore, the μβ_1907_ recorded at a lower concentration (−740 × 10^–48^ esu) remains constant within the experimental error of the EFISH technique (±15%),^17^ thus confirming the absence of aggregation in solution, in agreement with the UV–vis and ^1^H NMR spectroscopy evidence.

In conclusion, there is some indirect evidence suggesting a negative electronic third-order contribution to γ_EFISH_ as the origin of the negative value of the experimental γ_EFISH_ of **BP2**, **BP4**, **BP5**, and **BAP1**. In the case of **BP2**, **BP4**, and **BP5** the negative experimental value of γ_EFISH_ (Table 3) is a direct consequence of the rather low orientational dipolar contribution μ_0_β_λ_/5*kT*, in accordance with the low ground-state dipole moments (Table 4), so the negative electronic cubic term γ(−2ω; ω, ω, 0) prevails and produces a negative γ_EFISH_. Only when the dipole moment is high enough, as for **BP1** and particularly **BP3**, does the positive contribution of the orientational dipolar term predominate on γ(−2ω; ω, ω, 0), thus leading to positive γ_EFISH_ values.

Our interpretation is also confirmed by the negative experimental values of γ_EFISH_ for symmetric Zn^II^ porphyrins **BP4** and **BP5**. Indeed, they are characterized by dipole moments close to zero (Table 4)_,_ in agreement with their symmetrical structure, so the negative value of γ_EFISH_ is completely representative of that of the γ(−2ω; ω, ω, 0) third-order term.

It is worth stressing the relevant role of the steric hindrance characterizing the chromophores investigated in this work. For instance, in the case of asymmetric monosubstituted **BP2** carrying an ethynylphenyl linker with a donor pendant, we have a negative value of γ_EFISH_, which suggests a rather low second-order NLO response. However, this evidence is totally opposite to what reported in our previous investigation for a structurally related monosubstituted Zn^II^ porphyrin with simple phenyl groups in the 10,15,20-*meso*-positions, thus lacking the steric effects and electronic effects induced by more hindered and slightly donor 3,5-di-*tert*-butylphenyl groups.^4^

Therefore, the high EFISH second-order NLO response reported in that investigation^4^ could be tentatively explained by a J aggregation process in solution, due to the interaction between the donor −NR_2_ group of the substituent and the acid Zn^II^ center of another adjacent chromophore. In fact, J intermolecular aggregation is well-known to produce an increase of the second-order NLO response.^17^

## Conclusion

In this work we have reported the synthesis of a series of A_4_ β-pyrrolic-substituted Zn^II^ porphyrins, carrying one or two ethynylphenyl moieties with an electron-acceptor or -donor terminal group and bulky aryl substituents in the 5,10,15,20-*meso*-positions. Due to their sterically hindered architectures, these porphyrins are reasonably deemed to not show significant aggregation processes in solution. By a combined DFT and EFISH investigation, we produced clear evidence of the presence of charge transfer processes from the porphyrin core to the acetylenic fragment in the β-pyrrolic-position or vice versa, thus confirming the ambivalent donor or acceptor properties of the Zn^II^ porphyrin core suggested by some of us some years ago.^4^

Moreover, DFT calculations and the large differences of the EFISH second-order NLO responses of **BP1** and **BP2**, in the absence of significant aggregation processes, have shown that the A_4_ β-pyrrolic Zn^II^ porphyrins considered in this work behave better as donors than acceptors, in agreement also with the enhanced electron richness of the porphyrin core induced by the presence of 3,5-di-*tert*-butylphenyl groups in the 5,10,15,20-*meso*-positions.

The UV–vis electronic absorption spectra and the trend of the calculated dipole moments have confirmed that the introduction in β-pyrrolic-position of π-delocalized ethynylphenyl spacers equipped with either an acceptor or a donor group changes significantly the electronic properties of the porphyrin core. Furthermore, the two new Zn^II^ porphyrins, **BP3** and **BAP1**, have allowed us to highlight some relevant points, such as (i) the less facile electron transfer within the push–pull system involving the 2,12-β-pyrrolic-positions in comparison to the well-investigated push–pull system involving the 5,15-*meso*-positions and (ii) the noticeable effect of the introduction in the 5,10,15,20-*meso*-positions of the porphyrin core of four bulky donor bis(4-*tert*-butylphenyl)anilines, as in **BAP1**, leading to a substantially diminished involvement of the porphyrin core in the electron transfer process to the π-conjugated acceptor substituent in β position.

Finally, we have produced both theoretical and experimental evidence that in this kind of A_4_ β-pyrrolic substituted Zn^II^ porphyrins the electronic cubic contribution to γ_EFISH_, γ(−2ω; ω, ω, 0), cannot be neglected, if the polarity of the Zn^II^ porphyrin is so low that the dipolar orientational term to γ_EFISH_ becomes too small and if γ_EFISH_ and γ_THG_ are characterized by comparable values. In these particular cases, the determination of the second-order NLO properties by the EFISH technique may lead to overestimated or even negative values, if the purely electronic cubic contribution is neglected. As a general conclusion, our investigation proves that the determination of γ_EFISH_ for largely π-delocalized structures of low polarity must be carried out very carefully.

## Supporting Information Available

Synthetic procedures to obtain **BP1**–**5** and **BAP1**, their characterization, the ^1^H NMR spectra and the UV–vis spectra at different concentrations (PDF)
