# Supplementary material for: Electric-Field-Induced Second Harmonic Generation Nonlinear Optic Response of A4 β-Pyrrolic-Substituted ZnII Porphyrins: When Cubic Contributions Cannot Be Neglected
Source: Inorg Chem. 2020 May 15;59(11):7561–70. doi: 10.1021/acs.inorgchem.0c00451 (PMC8007098; doi:10.1021/acs.inorgchem.0c00451)
Supplement: Supplementary file 1 — ic0c00451_si_001.pdf [file ic0c00451_si_001.pdf]

Supporing information to

Electric-Field-Induced Second Harmonic Generation Nonlinear Optical response of A$_4$ β-pyrrolic substituted Zn^{II} porphyrins: When cubic contributions cannot be neglected

Gabriele Di Carlo,[a] Maddalena Pizzotti,[a] Stefania Righetto,[a] Alessandra Forni,*[b] Francesca Tessore.*[a]

[a]*Department of Chemistry, University of Milan, INSTM Research Unit, Via C. Golgi 19, 20133 Milano, Italy*

[b]*Istituto di Scienze e Tecnologie Chimiche "G. Natta" (SCITEC), Via C. Golgi 19, 20133 Milano, Italy*

corresponding author: francesca.tessore@unimi.it

co-corresponding author: alessandra.forni@scitec.cnr.it

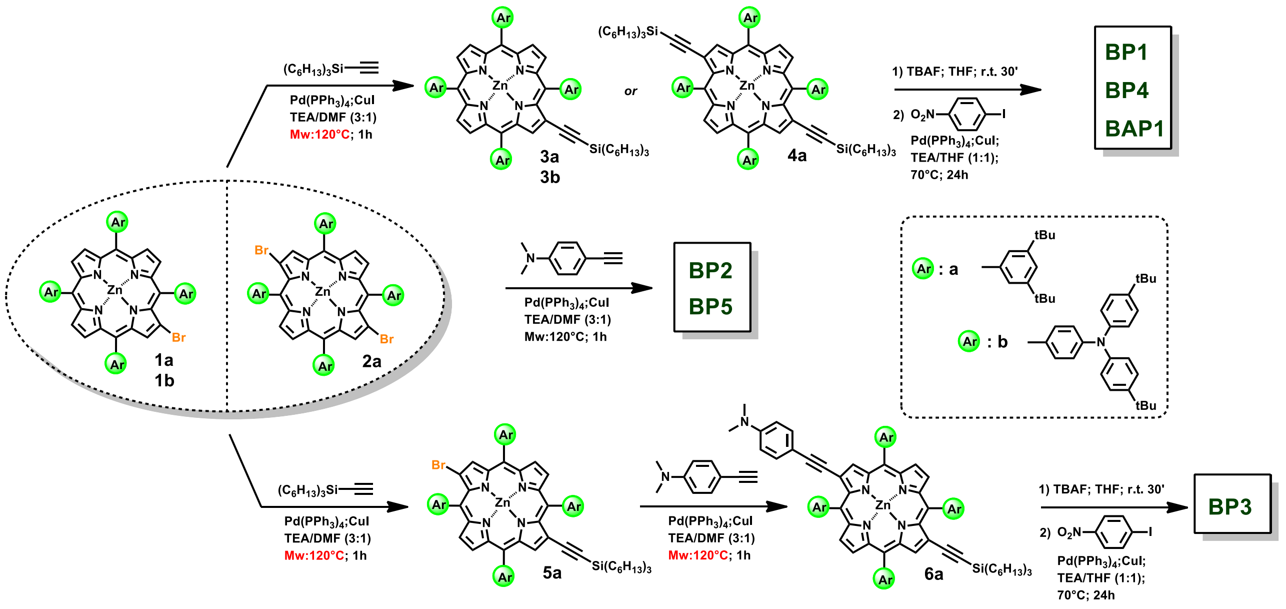

Scheme 1. Schematic synthetic procedure for BP1-5 and BAP1

General remarks

All the reagents and the solvents used in the syntheses were purchased from Sigma Aldrich and used as received, except NEt$_3$ (freshly distilled over KOH) and THF (freshly distilled from Na/benzophenone under nitrogen). Silica gel for gravimetric chromatography (Geduran Si 60, 63-

200 μm) and for flash chromatography (Kieselgel 60, 0.040-0.063 mm) were purchased from Merck. 2-Bromo-porphyrins (**1a** and **1b**) and 2,12-Dibromo-porphyrins (**2**) were prepared as reported in the literature.[1,2]

[1]*H*-NMR spectra were recorded on a Bruker Avance DRX-400 in pure $CDCl_3$ (*Cambridge Isotope Laboratories, Inc.*). Mass spectra were obtained with a VG Autospec M246 magnetic mass spectrometer with a LSIMS ionic source. Elemental analyses were carried out with a Perkin-Elmer CHN 2400 instrument in the Analytical Laboratories of the Department of Chemistry at the University of Milan. Electronic absorption spectra were recorded in CH_2Cl_2 solution at room temperature on a Shimadzu UV 3600 spectrophotometer.

General procedures of (Trihexylsilyl)acetylene insertion by assisted-microwave Sonogashira coupling reaction

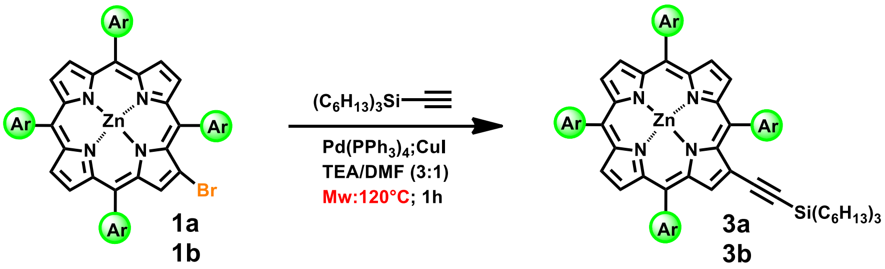

a) $Pd(PPh_3)_4$ (0.01 mmol), mono-bromo porphyrin (**1a** or **1b**; 0.10 mmol), freshly distilled NEt_3 (15mL), anhydrous DMF (5mL) and (Trihexylsilyl)acetylene (0.50 mmol) were introduced in a dry Schlenk tube and degassed with four freeze-pump-thaw cycles at -78°C. The mixture was transferred under dinitrogen flow in a microwave quartz vessel, and CuI (0.02 mmol) was then added. The mixture was allow to react in the microwave cavity at 120°C for 1 hr, after which the solvent was evaporated *in vacuo*. The crude was purified by flash chromatography using a mixture of *toluene/n-hexane* as eluent (15/85 for **3a** and 30/70 for **3b**) to give a green-purple solid.

3a (82% yield): ^{1}H-NMR (400 MHz, $CDCl_3$, 25°C) δ, ppm 9.31 (s, 1H), 9.00 (d, 4H), 8.94 (d, 1H), 8.70 (d, 1H), 8.11 (m, 6H), 8.02 (d, 2H), 7.82 (m, 4H), 1.57-1.53 (m, 72H), 1.41 (m, 12H), 1.32 (m, 12H), 0.90 (m, 9H), 0.67 (m, 6H).

3b (78% yield): ^{1}H-NMR (400 MHz, $CDCl_3$, 25°C) δ, ppm 9.38 (s, 1H), 9.12 (d, 4H), 9.07 (d, 1H), 8.90 (d, 1H), 8.10 (d, 6H), 7.95 (d, 2H), 7.49-7.47 (m, 24H), 7.49-7.37 (m, 16H), 1.44-1.40 (m, 96H), 1.34-1.26 (m, 9H), 0.88 (m, 6H).

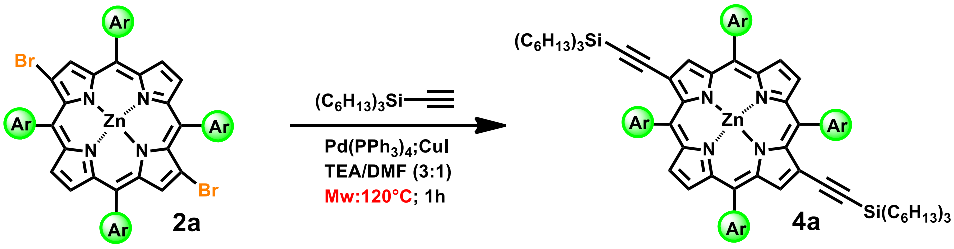

b) Pd(PPh3)4 (0.02 mmol), di-bromo porphyrin (**2a**; 0.10 mmol), freshly distilled NEt3 (12mL), anhydrous DMF (4mL) and (Trihexylsilyl)acetylene (1.00 mmol) were introduced in a dry Schlenk tube and degassed with four freeze-pump-thaw cycles at -78°C. The mixture was transferred under dinitrogen flow in a microwave quartz vessel, and CuI (0.03 mmol) was then added. The mixture was allow to react in the microwave cavity at 120°C for 1 h, after which the solvent was evaporated *in vacuo*. The crude was purified by flash chromatography using a mixture of *toluene/n-hexane* as eluent (20/80) to give a green-purple solid. (**4a**: 78% yield).

4a (78% yield): ^{1}H-NMR (400 MHz, $CDCl_3$, 25°C) δ, ppm 9.26 (d, 2H), 8.99 (s, 1H), 8.91 (d, 1H), 8.67 (d, 1H), 8.60 (s, 1H), 8.10 (dd, 4H), 7.99 (dd, 4H), 7.84-7.99 (m, 4H), 1.57 (d, 36H) , 1.54 (d, 36H), 1.40 (m, 24H), 1.31 (m, 24H), 0.90 (m, 18H), 0.67 (m, 12H).

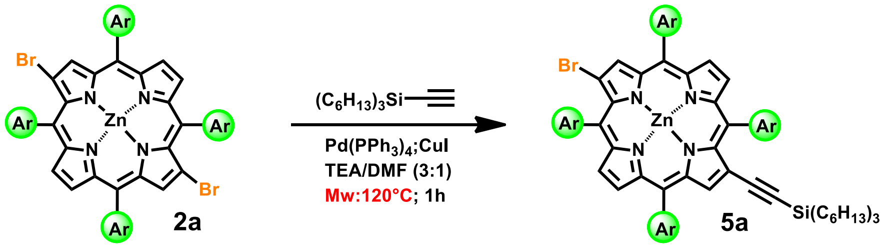

c) Pd(PPh3)4 (0.01 mmol), mono-bromo porphyrin (**2a**; 0.10 mmol), freshly distilled NEt3 (15mL), anhydrous DMF (5mL) and (Trihexylsilyl)acetylene (0.30 mmol) were introduced in a dry Schlenk tube and degassed with four freeze-pump-thaw cycles at -78°C. The mixture was transferred under dinitrogen flow in a microwave quartz vessel, and CuI (0.02 mmol) was then added. The mixture was allow to react in the microwave cavity at 120°C for 1 hr, after which the solvent was evaporated *in vacuo*. The crude was purified by flash chromatography using a mixture of *toluene/n-hexane* as eluent (20/80) to give a green-purple solid.

5a (38% yield): ^{1}H-NMR (400 MHz, $CDCl_3$, 25°C) δ, ppm 9.28 (s, 1H), 9.09 (s, 1H), 9.01-8.85 (m, 3H), 8.70-8.64 (m, 1H), 8.11 (s, 4H), 8.05 (dd, 4H), 8.00 (s, 4H), 7.93 (dd, 4H), 7.83-7.80 (m, 4H), 1.59-1.52 (m, 72H) , 1.41 (m, 12H), 1.31 (m, 12H), 0.90 (m, 9H), 0.67 (m, 6H).

General procedures of nitrobenzene terminal group insertion

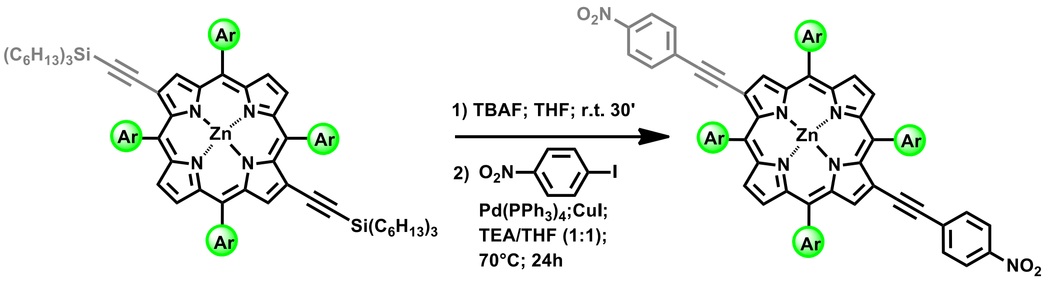

3a; 3b; 4a **BP1; BAP1;** BP4

0.05 mmol of selected trihexylsilyl derivative **3a**, **3b** or **4a** was dissolved under nitrogen in 10 mL of anhydrous THF, and then, 0.05 ml (0.10 ml for **4a**) of tetrabutylammonium fluoride (TBAF) solution 1.0 M in THF was dropwise added at 0°C. The mixture was allowed to react for 30 minutes at room temperature and then filtered through a silica plug to get, after evaporation of solvent, the pure acetylene deprotected intermediate. Immediately the proper intermediate was transferred in a dry Schlenk tube and dissolved under nitrogen in 1:1 mixture of freshly distilled NEt_3 and anhydrous THF. 1-iodo-4-nitrobenzene (0.1 mmol), $Pd(PPh_3)_4$ (0.001 mmol) and CuI (0.002 mmol) were then added and the resulting mixture degassed with four freeze-pump-thaw cycles at -78°C. The reaction mixture was finally warmed at 70°C and allowed to react for 24 hours then the solvent evaporated and the crude recovered with CH_2Cl_2. The organic phase was washed with water, dried over anhydrous Na_2SO_4 and after evaporation of solvent, the gravimetric chromatography purification of the crude using a mixture of CH_2Cl_2/*n-hexane* as eluent (30/70) and recrystallization at -20°C with CH_2Cl_2/MeOH provided the corresponding nitro-porphyrin as green solid.

BP1 (79% yield): ^{1}H-NMR (400 MHz, $CDCl_3$, 25°C) δ, ppm 9.36 (s, 1H), 9.00-8.94 (m, 5H), 8.73 (d, 1H), 8.21 (d, 2H), 8.12 (d, 4H), 8.09 (d, 4H), 7.85 (s, 1H), 7.80 (s, 3H), 7.47 (d, 2H), 1.56-1.53 (m, 54H), 1.45 (m, 18H).
MS-FAB(+) *m/z*: calcd for $C_{84}H_{95}N_5O_2Zn$ 1269, found 1270 $[M+H]^+$
Elemental analysis: calcd (%) C 79.31, H 7.53, N 5.51; found C 79.45, H 7.55, N 5.49.

BP4 (56% yield): ^{1}H-NMR (400 MHz, $CDCl_3$, 25°C) δ, ppm 9.33 (d, 2H), 8.94 (s, 1H), 8.89 (d, 1H), 8.71 (d, 1H), 8.67 (s, 1H), 8.21 (d, 4H), 8.11-8.07 (m, 8H), 7.86 (m, 2H), 7.82 (m, 2H), 7.46 (d, 4H), 1.57 (d, 36H) , 1.46 (d, 36H).
MS-FAB(+) *m/z*: calcd for $C_{92}H_{98}N_6O_4Zn$ 1415, found 1416 $[M+H]^+$
Elemental analysis: calcd (%) C 77.97, H 6.97, N 5.93; found C 77.77, H 6.95, N 5.95.

BAP1 (71% yield): ^{1}H-NMR (400 MHz, $CDCl_3$, 25°C) δ, ppm 9.45 (s, 1H), 9.05-8.90 (m, 6H), 8.32 (d, 2H), 8.05-7.96 (m, 8H), 7.85 (d, 2H), 7.48-7.29 (m, 36H), 7.02 (d, 4H), 1.42 (s, 54H), 1.38 (m, 18H).

MS-FAB(+) *m/z*: calcd for $C_{132}H_{131}N_9O_2Zn$ 1938, found 1939 $[M+H]^+$

Elemental analysis: calcd (%) C 81.68, H 6.80, N 6.49; found C 81.87, H 6.78, N 6.51.

General procedures of N,N-dimethylaniline terminal group insertion

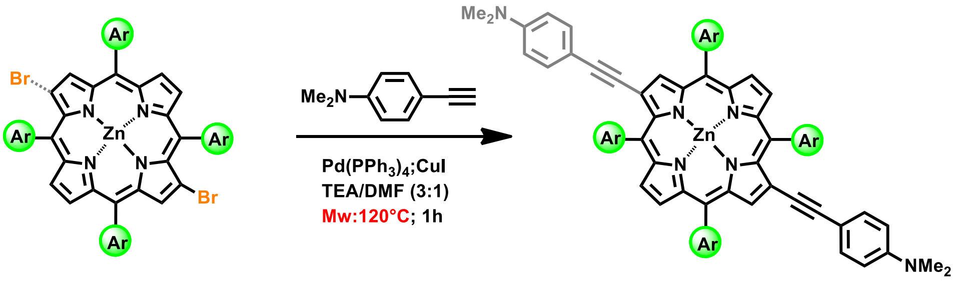

1a; 2a   **BP2**; BP5

$Pd(PPh_3)_4$ (0.02 mmol), mono-bromo porphyrin (**1a**; 0.20 mmol), or di-bromo porphyrin (**2a**; 0.10 mmol), freshly distilled NEt_3 (15mL), anhydrous DMF (5mL) and 4-Ethynyl-*N,N*-dimethylaniline (1.0 mmol) were introduced in a dry Schlenk tube and degassed with four freeze-pump-thaw cycles at -78°C. The mixture was transferred under dinitrogen flow in a microwave quartz vessel, and CuI (0.03 mmol) was then added. The mixture was allow to react in the microwave cavity at 120°C for 1 hr, after which the solvent was evaporated *in vacuo*. The crude was then purified by flash chromatography by using a mixture of CH_2Cl_2/*n-hexane* as eluent (30/70 for **BP2** and 65/35 for **BP5**) and the following recrystallization at -20°C with CH_2Cl_2/MeOH provided the corresponding amino-porphyrin as dark-green solid (**BP2**: 46% yield; **BP5**: 40% yield;).

BP2 (46% yield): ^{1}H-NMR (400 MHz, $CDCl_3$ +Py-d_5 25°C) δ, ppm 9.19 (s, 1H), 8.90 (s, 2H), 8.85-8.82 (m, 3H), 8.66 (d, 1H), 8.07-8.04 (m, 10H), 7.79 (d, 4H), 7.23 (d, 2H), 6.5 (d, 2H), 3.04 (s, 6H), 1.55-1.50 (m, 54H), 1.47 (d, 18H).

MS-FAB(+) *m/z*: calcd for $C_{86}H_{101}N_5Zn$ 1268, found 1269 $[M+H]^+$

Elemental analysis: calcd (%) C 81.32, H 8.02, N 5.51; found C 81.48, H 7.99, N 5.53.

BP5 (40% yield): ^{1}H-NMR (400 MHz, $CDCl_3$, 25°C) δ, ppm 9.20 (d, 2H), 8.90 (s, 1H), 8.85 (d, 1H), 8.71 (d, 1H), 8.66 (s, 1H), 8.07 (s, 8H), 7.81 (s, 4H), 7.23 (d, 2H), 6.69 (d, 2H), 3.05 (s, 12H), 1.54 (d, 36H), 1.46 (d, 36H).

MS-FAB(+) *m/z*: calcd for $C_{96}H_{110}N_6Zn$ 1411, found 1412 $[M+H]^+$

Elemental analysis: calcd (%) C 81.58, H 7.84, N 5.95; found C 81.88, H 7.82, N 5.96.

General procedures of BP3 synthesis

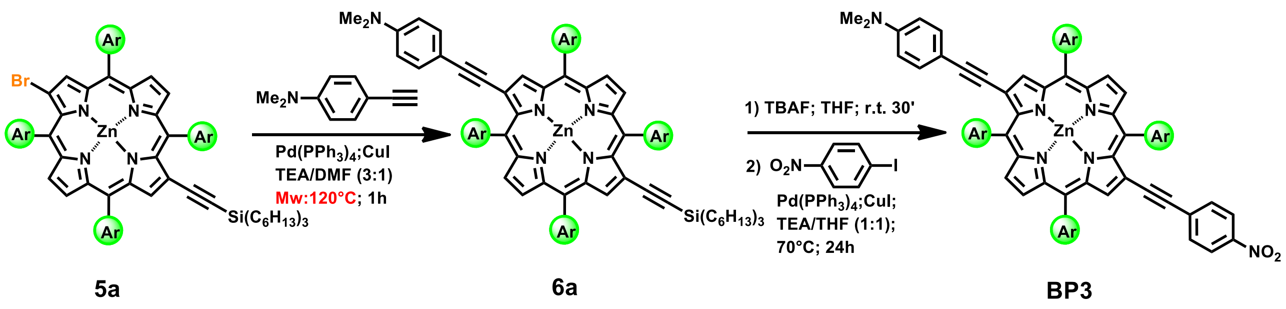

0.05 mmol of the bromo derivative **5a** was allowed to react with 0.15 mmol of 4-Ethynyl-*N*,*N*-dimethylaniline to get **6a** (43% yield) by following the procedure formerly described for **BP2**.

The purified trihexylsilyl intermediate **6a** was subsequently deprotected with TBAF and allowed to react with 15 mmol of 1-iodo-4-nitrobenzene as for the previously described **BP1**.

6a: ^{1}H-NMR (400 MHz, $CDCl_3$, 25°C) δ, ppm 9.26 (s, 1H), 9.21 (d, 1H), 8.98-8.84 (m, 2H), 8.74-8.60 (m, 2H), 8.09 (s, 4H), 8.07 (s, 2H), 7.99 (s, 2H), 7.82 (s, 3H), 7.79 (s, 1H), 7.23 (d, 2H), 6.66 (d, 2H), 3.02 (s, 6H), 1.57 (m, 36H), 1.54 (m, 18H), 1.47 (d, 18H), 1.40 (m, 12H), 1.31 (m, 12H), 0.90 (m, 9H), 0.67 (m, 6H).

BP3: ^{1}H-NMR (400 MHz, $CDCl_3$, 25°C) δ, ppm 9.32 (s, 1H), 9.21 (d, 1H), 8.92 (s, 1H), 8.87 (m, 1H), 8.73-8.64 (m, 2H), 8.20 (d, 2H), 8.11-8.07 (m, 8H), 7.83 (m, 3H), 7.80 (s, 1H), 7.47 (d, 2H), 7.22 (d, 2H), 6.64 (d, 2H), 3.02 (s, 6H), 1.56 (m, 36H), 1.48 (m, 18H), 1.45 (m, 18H).

MS-FAB(+) *m/z*: calcd for $C_{94}H_{104}N_6O_2Zn$ 1412, found 1413 $[M+H]^+$

Elemental analysis: calcd (%) C 79.77, H 7.41, N 5.94; found C 79.56, H 7.40, N 5.96.

^{1}H-NMR spectra of BP1-5 and BAP1

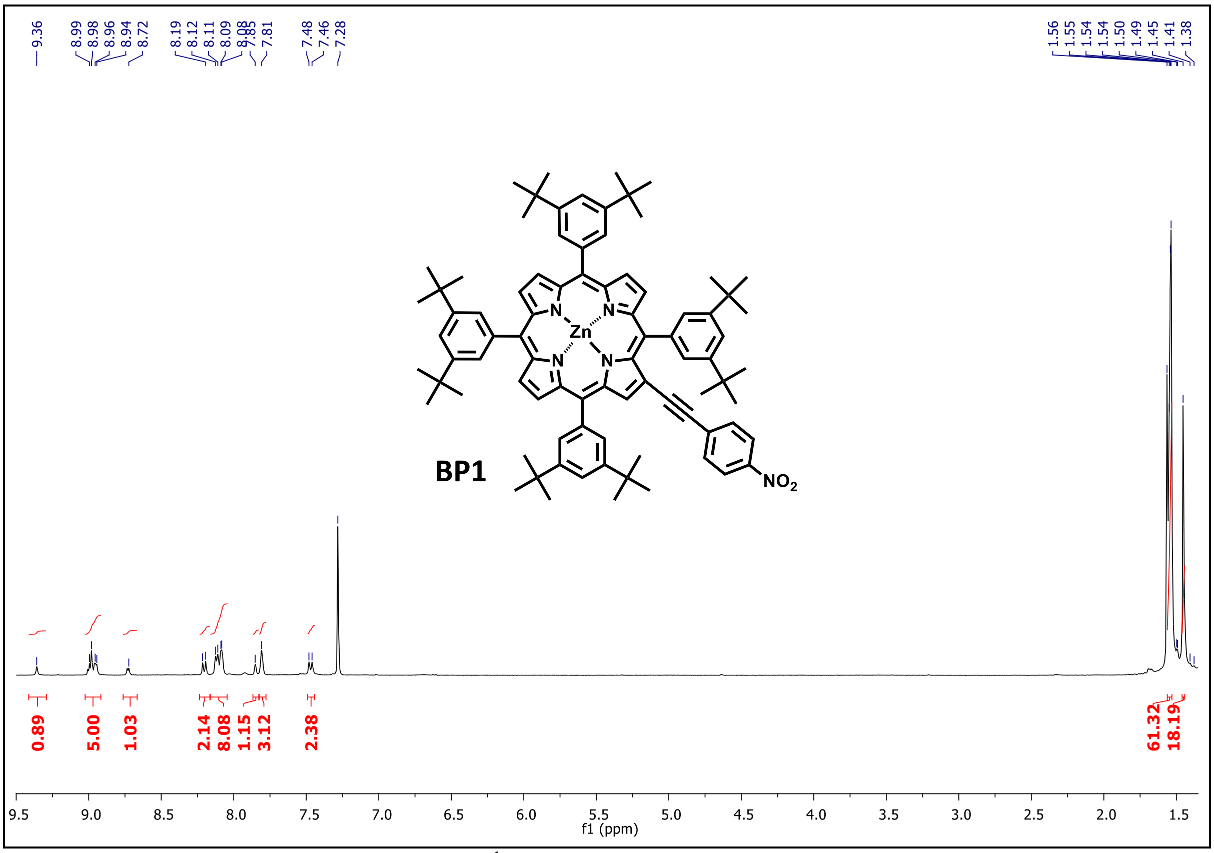

Figure S1. ^{1}*H*-NMR spectrum of **BP1** in $CDCl_3$.

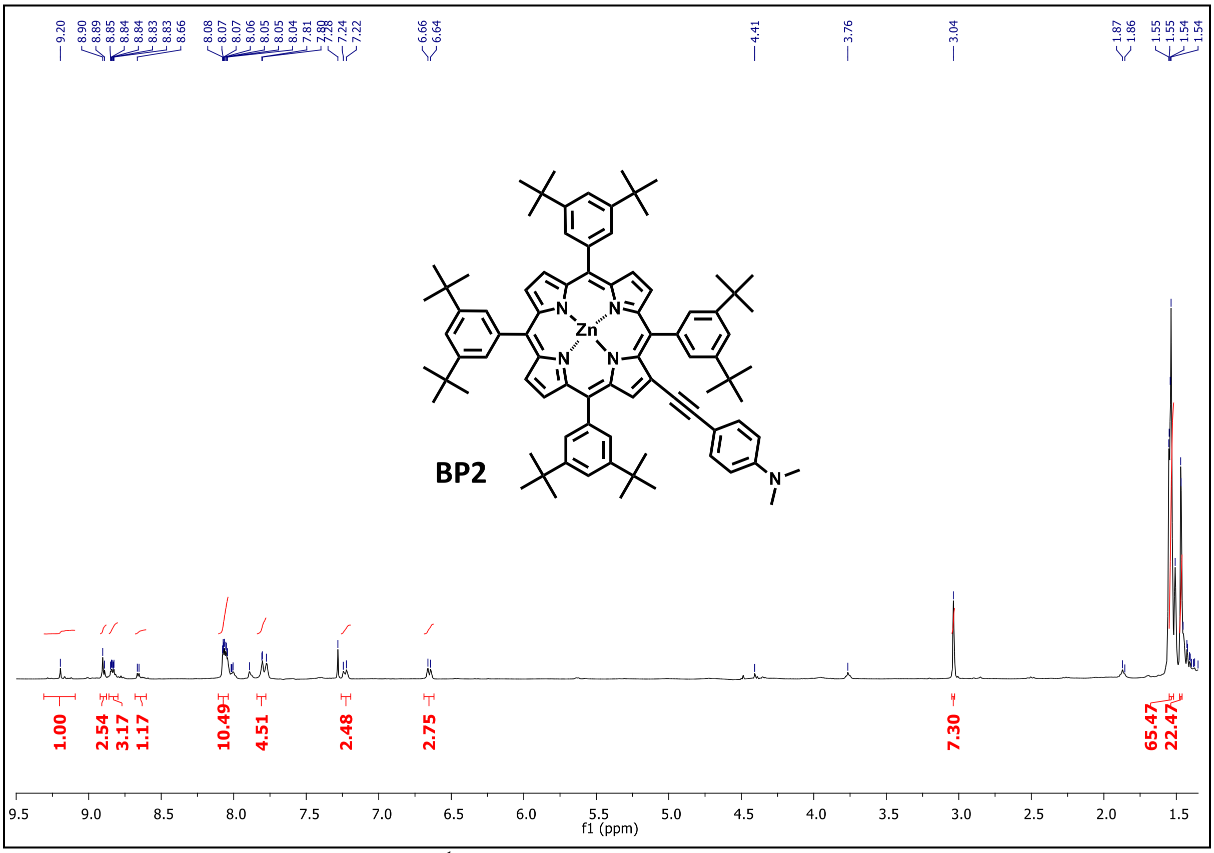

Figure S2. ^{1}*H*-NMR spectrum of **BP2** in $CDCl_3$.

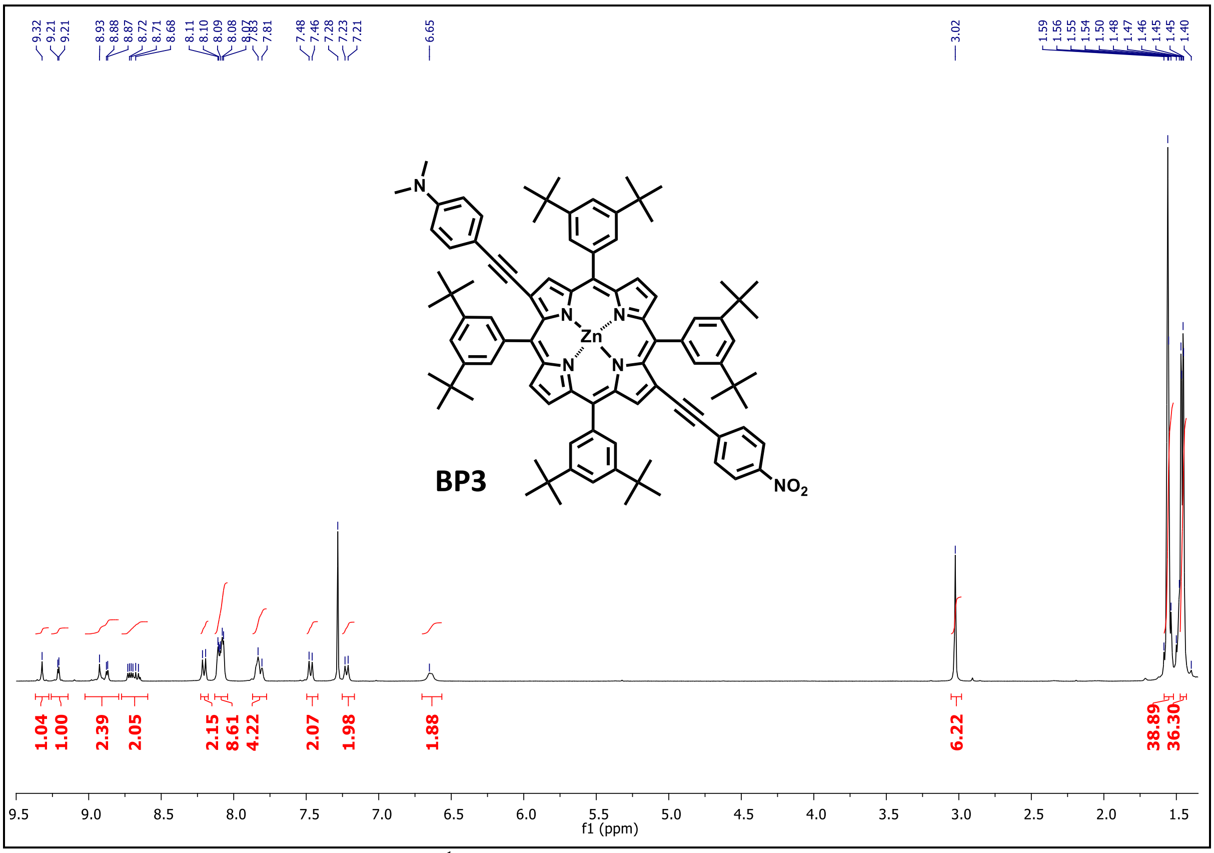

Figure S3. ^{1}H-NMR spectrum of **BP3** in $CDCl_3$.

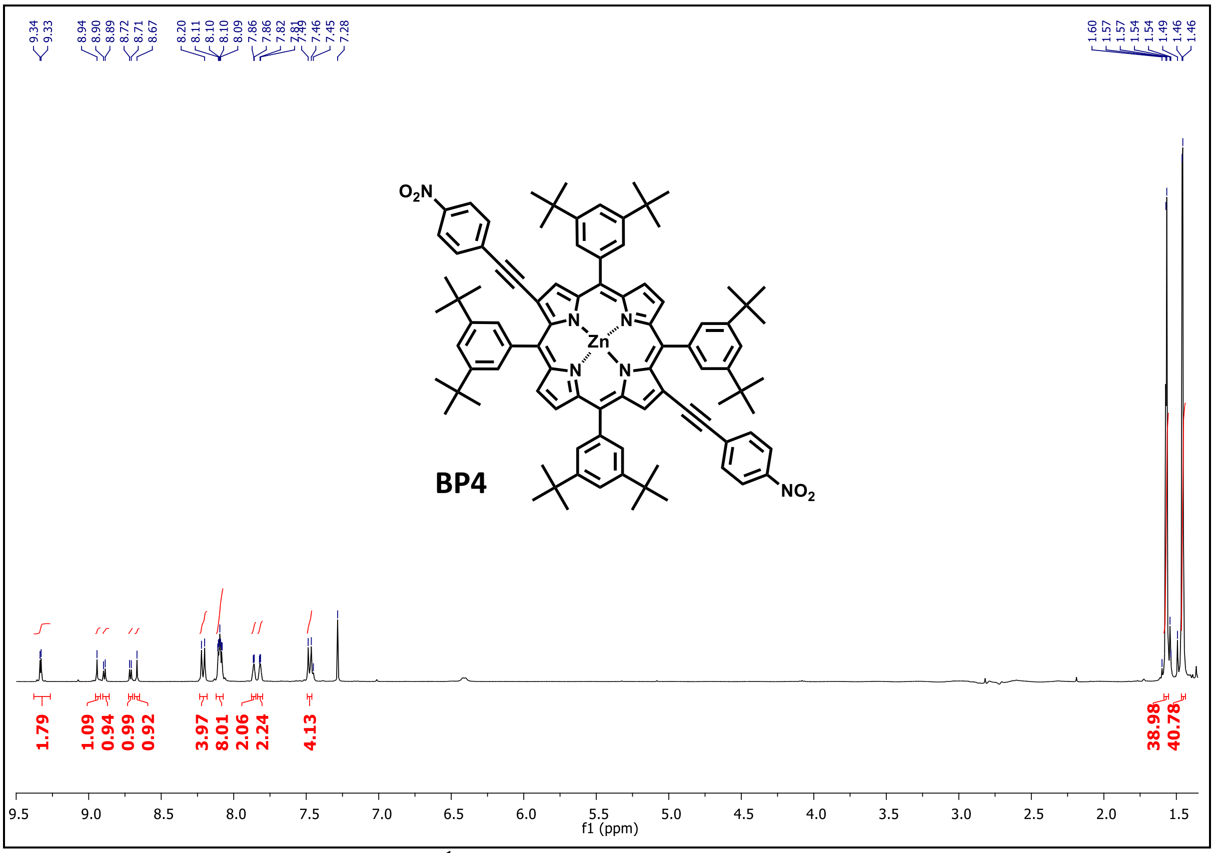

Figure S4. ^{1}H-NMR spectrum of **BP4** in $CDCl_3$.

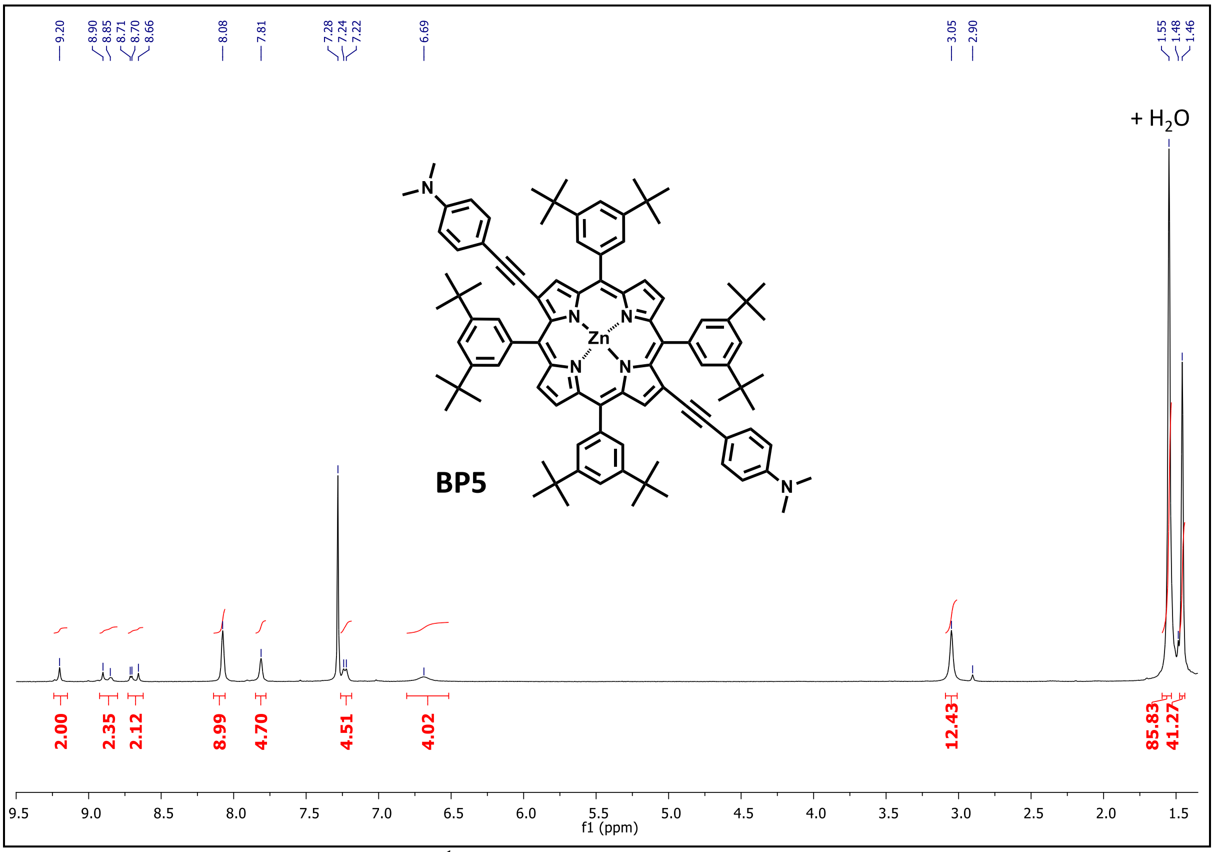

Figure S5. *^{1}H*-NMR spectrum of **BP5** in $CDCl_3$.

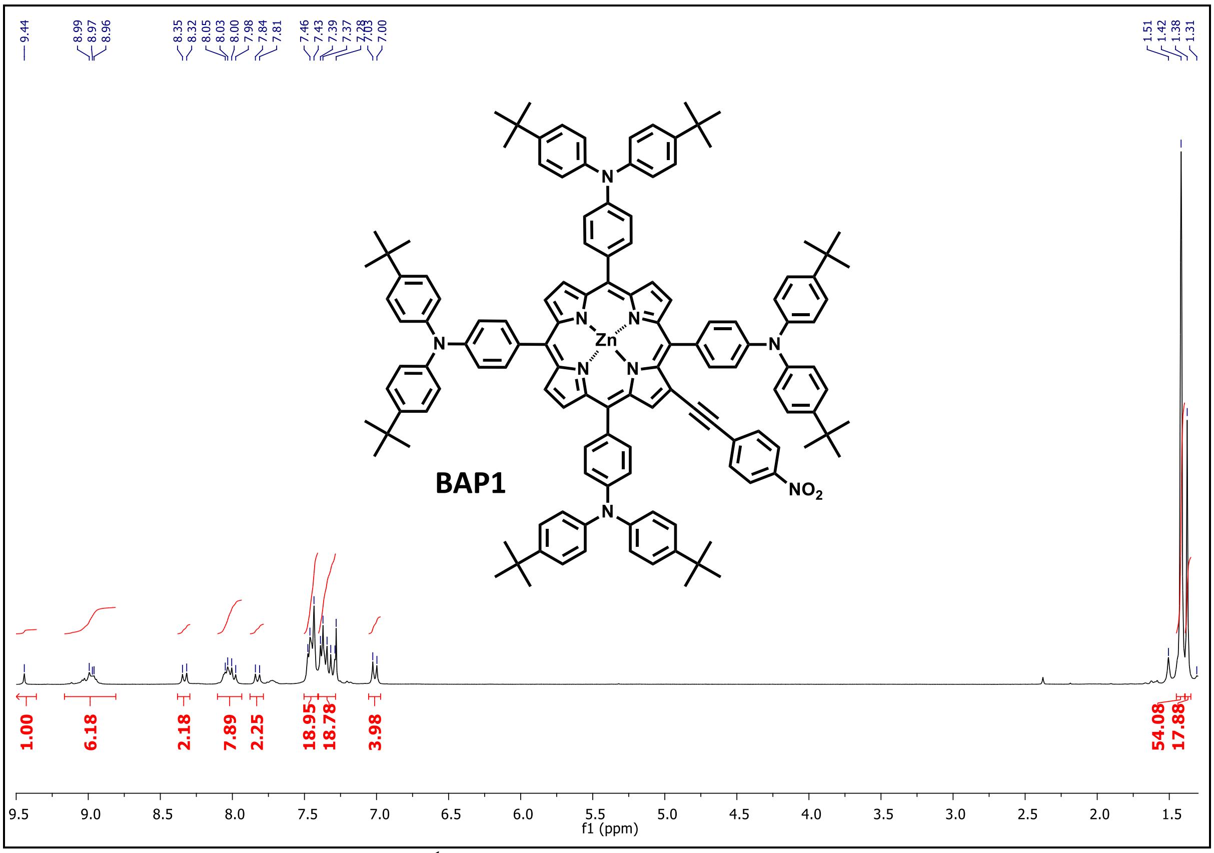

Figure S6. *^{1}H*-NMR spectrum of **BAP1** in $CDCl_3$.

UV-Vis absorption spectra of BP1-5 and BAP1in CH_2Cl_2 at various concentrations

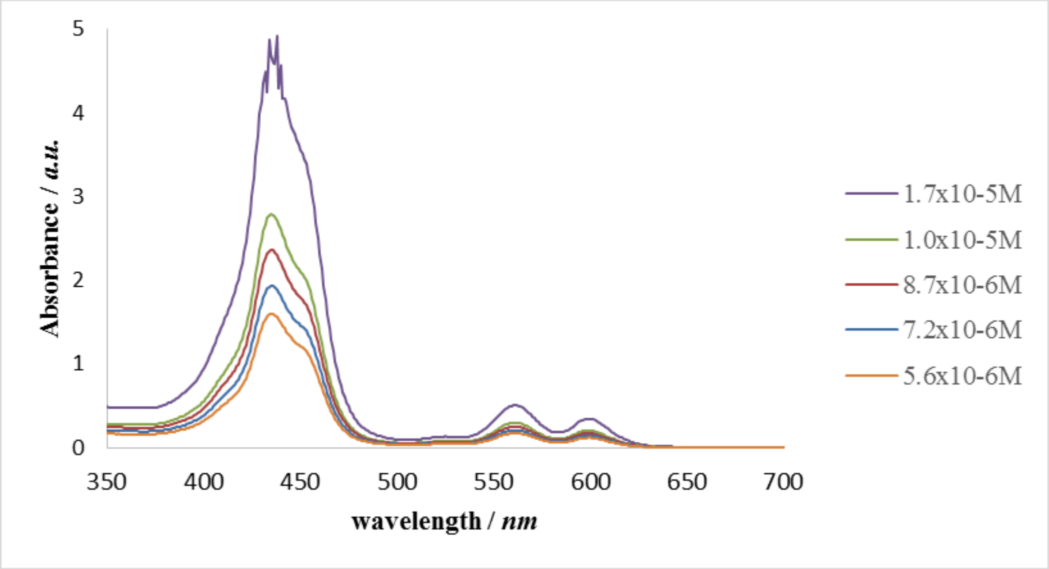

Figure S7. Uv-Vis absorption spectrum of BP1 at different concentrations

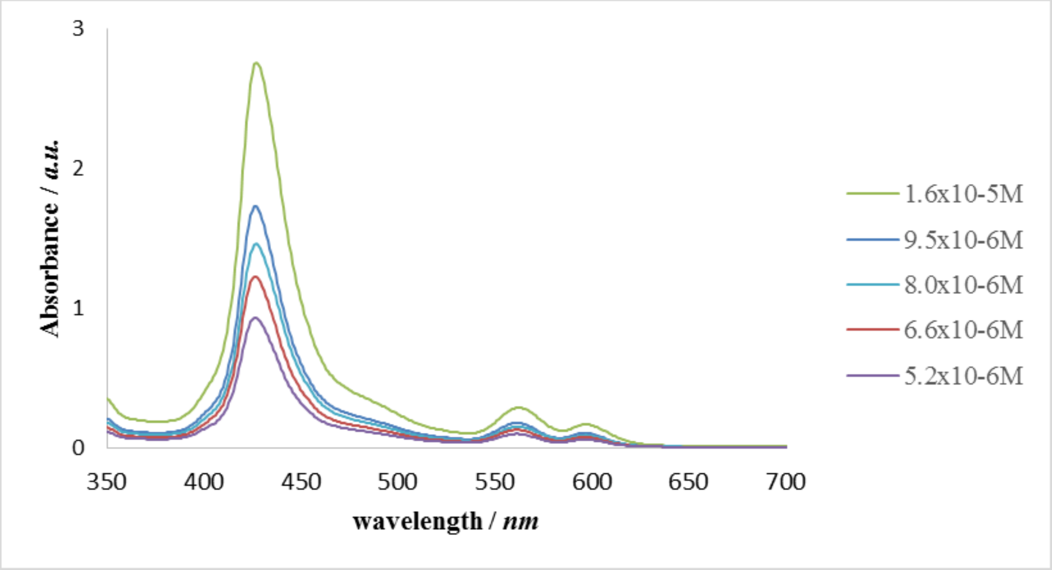

Figure S8. Uv-Vis absorption spectrum of BP2 at different concentrations

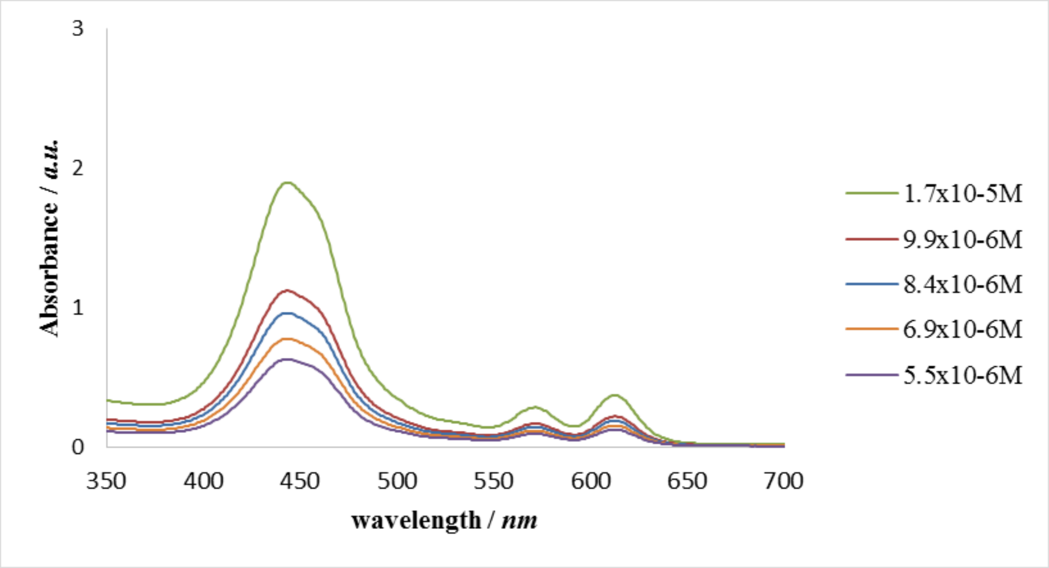

Figure S9. Uv-Vis absorption spectrum of BP3 at different concentrations

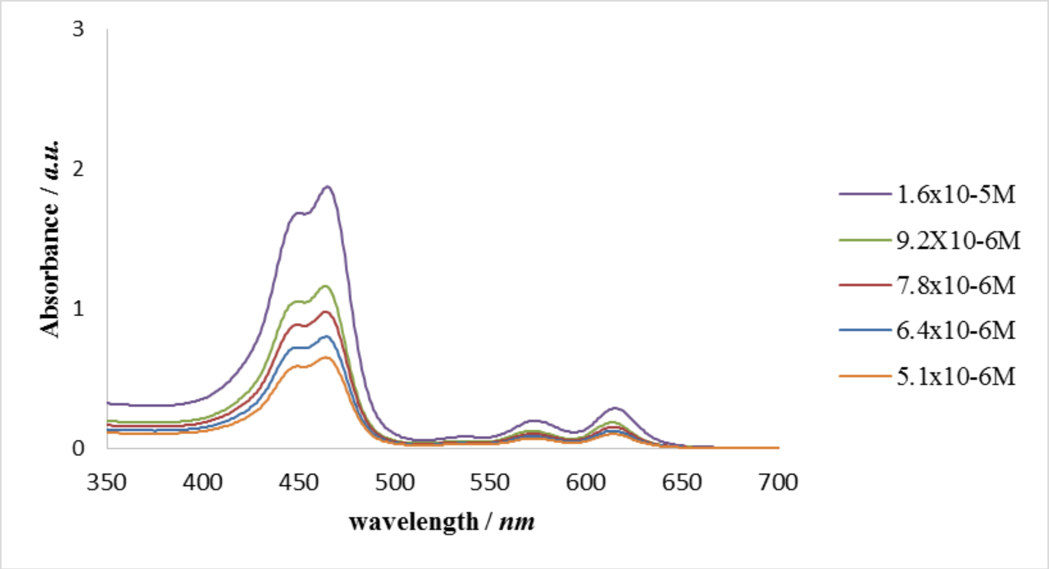

Figure S10. Uv-Vis absorption spectrum of BP4 at different concentrations

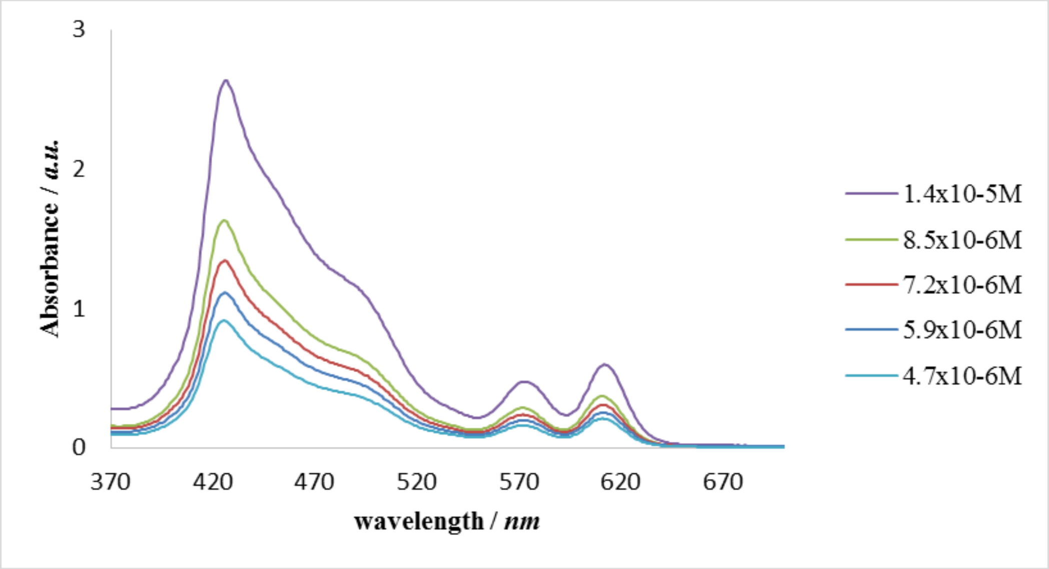

Figure S11. Uv-Vis absorption spectrum of BP5 at different concentrations

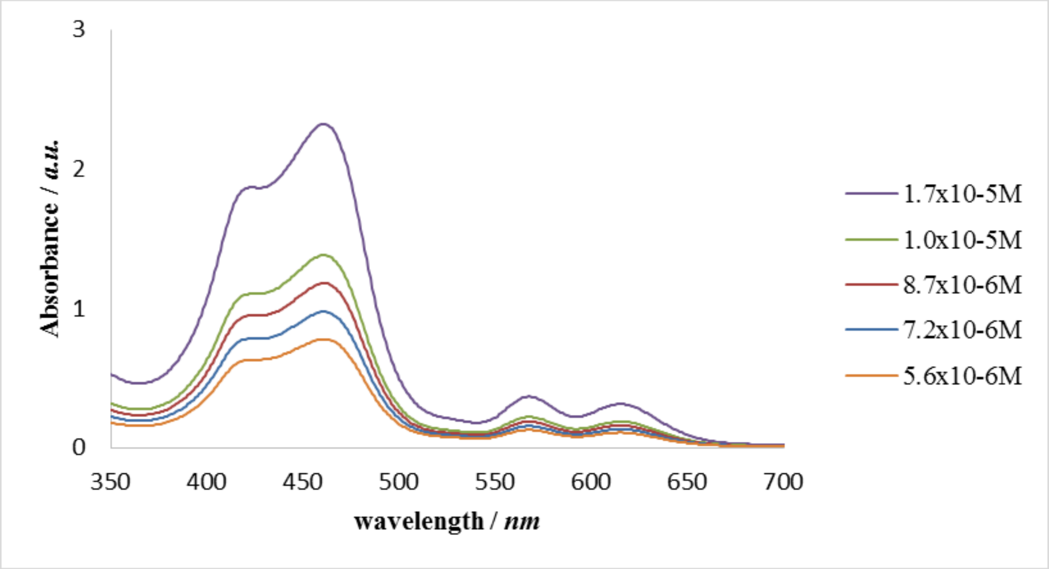

Figure S12. Uv-Vis absorption spectrum of BAP1 at different concentrations

References

1) Di Carlo, G.; Orbelli Biroli, A.; Tessore, F.; Rizzato, S.; Forni, A.; Magnano, G.; Pizzotti, M. Light-induced regiospecific bromination of *meso*- tetra(3,5-di-*tert*-butylphenyl)porphyrin on 2,12 β-pyrrolic positions. *J. Org. Chem.* **2015**, *80*, 4973-4980.

2) Covezzi, A.; Orbelli Biroli, A.; Tessore, F.; Forni, A.; Marinotto, D.; Biagini, P.; Di Carlo, G.; Pizzotti, M. 4D–π–1A type β-substituted Zn^{II}-porphyrins: ideal green sensitizers for building-integrated photovoltaics. *Chem. Commun*. **2016**, *52*, 12642-12645.
